# The role of ATG16L1 in Crohn’s disease and the structural alteration mechanisms and functional consequences of the rs2241880 variant

**DOI:** 10.3389/fmed.2025.1656575

**Published:** 2025-10-03

**Authors:** Shijie Ren, Wenli Wei, Xingchi Liu, Xin Kang, Jingyuan Liu, Wenjing Zhai, Yifan Zhang, Qiang Chuai, Boqian Hu, Jianping Liu, Xiaomeng Lang

**Affiliations:** ^1^Graduate School, Hebei University of Chinese Medicine, Shijiazhuang, Hebei, China; ^2^Department of Gastroenterology, The First Affiliated Hospital of Hebei University of Chinese Medicine, Shijiazhuang, Hebei, China

**Keywords:** AI modeling, AlphaFold3, ATG16L1, missense mutation, molecular dynamics, structural prediction

## Abstract

**Background:**

ATG16L1 (Autophagy Related 16 Like 1) is a key regulatory protein in the autophagy pathway. Although previous studies have established a significant association between the ATG16L1 genotype and Crohn’s disease (CD) susceptibility, the specific molecular mechanism of its high-frequency missense variant rs2241880 has yet to be systematically elucidated.

**Methods:**

In this study, we first confirmed the important role of ATG16L1 in CD pathogenesis through genome-wide association study analysis and Western blot, as well as qRT-PCR. Subsequently, high-precision structural prediction, protein model-based dynamic simulation, and AI model thermodynamic stability analysis were innovatively integrated. The thermal shift assay (TSA) was employed to validate the structural stability of the mutant, while the pull-down assay was used to examine its binding capacity with WIPI2b.

**Results:**

The results show that ATG16L1 plays a significant role in the pathogenesis of CD. The mutation causes the protein’s overall conformation to become more compact, significantly increasing the rigidity of key functional regions, and enhancing the structural and thermodynamic stability, which in turn affects the cleavage efficiency of caspase-3 and the function of the WD40 domain. The results of the TSA experiment provided evidence for the computational findings. The pull-down assay confirmed that the binding capacity of the mutant to WIPI2b was significantly impaired.

**Conclusion:**

This finding not only provides the first molecular mechanism of the ATG16L1 T300A mutation, offering an important theoretical basis for understanding CD susceptibility differences, but also provides insights for precision medicine interventions and gene editing strategies.

## 1 Introduction

The rapid development of Artificial Intelligence (AI) technology is profoundly reshaping the research paradigm in life sciences. Deep learning and machine learning models have become core tools in this field, particularly showing a profound impact in key areas such as protein structure prediction, genomics analysis, and drug development (1). In recent years, AI technology has brought about major transformations in biological research methodologies (2). In the field of protein research, AI models have made milestone advancements: the long-standing protein folding problem has been substantially resolved due to breakthroughs from models like AlphaFold; the accuracy of protein 3D structure prediction and mutation functional analysis has greatly improved (3).

ATG16L1 (Autophagy Related 16 Like 1) is one of the core regulatory proteins in the autophagy pathway, involved in the formation of autophagosomes, and plays a key role in clearing intracellular pathogens and maintaining intestinal epithelial barrier homeostasis (4). The function of ATG16L1 is closely related to Crohn’s disease (CD) susceptibility. Studies have shown that defects in its expression in Paneth cells and intestinal epithelial cells significantly exacerbate intestinal inflammation (5). The 300th site of ATG16L1 (rs2241880), a key mutation site in CD research, causes a missense mutation. This mutation is thought to potentially affect the stability of the ATG16L1 protein, weaken its binding with partner proteins, thereby impairing autophagosome formation ability (6).

However, despite extensive research on the relationship between ATG16L1 and CD, the mechanistic analysis of how the ATG16L1 300th site mutation specifically affects the protein’s structure and function remains insufficient. Currently, research on ATG16L1 mainly focuses on the relationship between genotype and phenotype, especially through phenotype analysis and genetic association studies to reveal the link between mutations and CD (7). However, there is still inadequate exploration of how mutations affect the stability of the ATG16L1 protein at the molecular level, particularly in terms of protein 3D structure, folding dynamics, and functional changes. Detailed mechanistic analyses are lacking in these aspects. Therefore, it is crucial to conduct in-depth studies on the mutation at the ATG16L1 300th site and reveal its molecular mechanism in protein stability and functional loss. With the introduction of AI technologies, particularly models like AlphaFold3 and ThermoMPNN, we now have new research tools at our disposal. By integrating these AI tools, we can delve into the molecular-level effects of the ATG16L1 300th site mutation on protein structure and further uncover its potential mechanisms in CD. The thermal shift assay (TSA) and pull-down assay can be employed to further validate the computational results.

## 2 Materials and methods

### 2.1 GWAS analysis

#### 2.1.1 The GWAS data source for CD

The GWAS data for CD comes from summary data in the IEU database (ieu-a-30), which includes 5,956 CD patients. The control group consists of 14,927 healthy individuals of European ancestry, matched with the case group in terms of age, sex, and geographical location, covering both males and females. For detailed information on ethical approval and informed consent, please refer to the published paper (8).

#### 2.1.2 Gene annotation

MAGMA evaluated the overall impact of a gene or genomic region by integrating the effects of multiple single nucleotide polymorphisms (SNPs) (±10 kb), rather than considering the effect of each SNP separately (9). The software uses 1,000 Genomes European phase 3 LD data (10). Building on this, gene set analysis based on MAGMA was used to analyze pathways associated with the pathogenic causes of CD. MAGMA aggregated the effects of multiple SNPs at the gene level, and after obtaining the effect estimates for each gene, it organized these genes into predefined gene sets, while also calculating the overall effect size of each gene set. Pathways from KEGG, BioCarta, and Reactome, necessary for the software, were obtained from the database at https://www.gsea-msigdb.org/gsea/msigdb (11).

#### 2.1.3 TWAS analysis

Risk genes were identified via gene annotation and TWAS, using UTMOST and FUSION. The study integrated GTExV8 eQTL (49 tissues) and CD GWAS data to explore tissue-specific genetic variants. UTMOST performed single-tissue TWAS followed by cross-tissue analysis with a multivariate model accounting for tissue-specific eQTL effects (11). FUSION, using GTExV8 whole blood eQTL and CD GWAS data, built penalized linear models with 500-kb cis windows to validate findings. Both analyses applied Benjamini-Hochberg correction, defining significance at false discovery rate (FDR) < 0.05.

#### 2.1.4 Conditional and conjoint analysis

Conditional and joint analysis of TWAS signals (FDR-adjusted) identified chromosomal key SNPs. Using CD GWAS summary statistics and 1,000 Genomes European LD reference (12), the analysis removed TWAS signals via conditional modeling, reapplied FDR correction, selected SNPs at *P*_FDR_ < 0.05, and evaluated combined effects post-optimization.

#### 2.1.5 Precise localization of risk genes

FOCUS fine-mapped CD GWAS data to risk regions using summary statistics, eQTL weights, and linkage disequilibrium (13). It evaluates gene sets’ roles in TWAS signals and genomic risk. Using GTExV8 weights, significant genes were defined by posterior inclusion probability (PIP) ≥ 0.8 and *p* < 5e^−8^.

#### 2.1.6 Intersection and colocalization analysis of gene analysis results

Key genes were identified by intersecting risk genes from gene annotation, cross-tissue TWAS, single-tissue TWAS, and fine-mapping analysis, followed by colocalization analysis. The “coloc” R package (14, 15) was utilized for colocalization analysis to assess the overlap between GWAS and eQTL signals at causal variant sites.

### 2.2 Validation of animal experiments

#### 2.2.1 Animal modeling

The experimental animals were housed at Hebei Provincial Hospital of Traditional Chinese Medicine, with approval from the Ethics Committee (IACUC-HPHCM-2024037). Colitis was induced using TNBS (Sigma-Aldrich). A 5% TNBS solution was mixed with absolute ethanol at a 1:1 volume ratio to prepare a 50% ethanol solution containing 2.5 mg/mL TNBS. The rats were administered an enema at a dose of 100 mg/kg body weight at a depth of 8 cm proximal to the anus and maintained in a head-down position for approximately 30 s to ensure the mixture reached the entire colon. Control group: received an enema with an equal volume of 50% ethanol. The modeling period lasted for 7 days.

#### 2.2.2 qRT-PCR

Total RNA was extracted from rat colon tissue following the manufacturer’s instructions for the RNA extraction kit. The mRNA was then reverse transcribed into cDNA using reverse transcriptase. The reaction conditions were followed, and amplification was performed on a fluorescent quantitative PCR machine, completing a total of 40 cycles. *β*-actin was used as the internal reference, and mRNA expression was analyzed using the 2^−ΔΔCt^ method.

#### 2.2.3 Western blot

Rat colon tissue was minced, and protein content was measured according to the instructions of the protein extraction kit. The protein samples were boiled for 5 min to denature, transferred to a membrane, and then incubated in 5% skim milk on a shaker for 2 h to block. The membrane was incubated overnight at 4 °C with a primary antibody against ATG16L1 (1:800). After four washes with TBST, a secondary antibody (1:8,000) was added, and incubation was carried out at room temperature for 1.5 h, followed by four additional washes with TBST. The membrane was placed in an exposure box and exposed in a dark room. After developing, fixing, and scanning the images, the brightness values of the protein bands from each group were analyzed. The corrected protein band brightness value (the ratio of each sample’s protein band brightness value to the internal reference band *β*-actin brightness value) was calculated. The control group was used as the standard for normalization.

### 2.3 AI modeling and prediction analysis

#### 2.3.1 Identification of protein amino acid mutation sites corresponding to missense mutation

In this study, we analyzed the rs2241880 mutation in ATG16L1. This gene has been confirmed by multiple studies to be closely associated with CD (16). To clarify the specific impact of the mutation on the ATG16L1 protein sequence, we used the NCBI Gene^1^ and Ensembl^2^ databases to query detailed gene annotations. Using the previous strategy, we queried the rs2241880 missense mutation on ATG16L1 and conducted further site searches using the Ensembl database (17, 18). The primary focus was on the amino acid change caused by the missense mutation.

#### 2.3.2 Acquisition of ATG16L1 wild type structure and download of amino acid sequence

To perform subsequent modeling and simulations, we needed to obtain the structure and amino acid sequence of the wild type ATG16L1 protein (Supplementary Table S1). To achieve this, we accessed the GeneCards and UniProt databases to gather detailed information and related data on ATG16L1.

##### 2.3.2.1 Querying ATG16L1 gene information through GeneCards

We first queried the detailed information of the ATG16L1 gene through the GeneCards database.^3^ From the query, we obtained the UniProt ID for ATG16L1: Q676U5.

##### 2.3.2.2 Downloading structure and sequence through UniProt

Next, based on the obtained UniProt ID and selecting the species as human, we searched and downloaded the complete amino acid sequence of ATG16L1 through the UniProt database.^4^ In UniProt, we found that the structure of this protein was publicly available and could be downloaded directly (19).

#### 2.3.3 ATG16L1 mutant AI modeling based on AlphaFold3

We chose to use AlphaFold3 for protein structure modeling of the mutant. AlphaFold3 utilizes an enhanced deep learning model that combines amino acid sequences, evolutionary information, and physicochemical knowledge to predict the three-dimensional structure of proteins with high precision (20). Compared to traditional experimental methods (such as X-ray crystallography and nuclear magnetic resonance), AlphaFold3 provides faster predictions with higher accuracy, especially when handling complex protein mutations, allowing for better simulation of the impact of mutations on protein structure (21).

We submitted the amino acid sequence of the ATG16L1 mutant to the AlphaFold3 online platform.^5^ After submission, AlphaFold3 automatically predicted the structure and generated a 3D model of the protein. We downloaded the CIF (crystallographic information file) and used PyMOL 3.2 educational edition to convert the file to PDB (protein data bank) format.

#### 2.3.4 *In vivo* protein simulation of ATG16L1

After obtaining the 3D structures of the wild type and mutant ATG16L1 proteins, we used GROMACS 2024.5 to perform molecular dynamics simulations to simulate the dynamic behavior and stability of the ATG16L1 protein in solution. The specific steps of the molecular dynamics simulation in this study are as follows:

##### 2.3.4.1 Hardware and software configuration for *in vivo* protein simulation

We used GROMACS 2024.5 for the molecular dynamics simulations. The parallel computing capabilities of GROMACS make it highly suitable for efficiently handling large-scale biomolecular simulations (22). Our simulations ran on the Ubuntu 24.04 LTS operating system.

Simulation parameter settings: force field and water model: we selected the AMBER14SB force field for the parameterization of ATG16L1. The AMBER14SB force field is commonly used in protein simulations and accurately describes the interactions between amino acid residues in proteins (23). Solvent model: we chose the TIP3P water model, a classical water molecule model suitable for describing the behavior of water molecules in biomolecular solutions (24). System construction and solvation: The pdb2gmx tool was used to generate the protein topology file, and the editconf tool was used to place the protein in a cubic box, with the minimum distance between the box and the protein set to 1.0 nm. We then used the solvate command for solvation, ensuring that water molecules were evenly distributed and filled the gaps. Ionization: the genion tool was used to add Na+ and Cl− ions to ensure the system’s electro-neutrality and simulate the ion concentration under physiological conditions (0.15 M NaCl). Energy minimization and equilibration: we performed 1,000 steps of energy minimization using the steepest descent algorithm, with the maximum step size set to 0.01 nm, to eliminate unreasonable contacts and structures in the system. Equilibration process: the system was first equilibrated for 100 ps under constant temperature and volume conditions, followed by 100 ps equilibration under constant temperature and pressure conditions to ensure system stability. Mutant protein dynamics simulation: after equilibration, we performed a 100 ns production molecular dynamics simulation under constant temperature and pressure conditions with a temperature of 300 K and pressure of 1.0 atm. The time step was 2 fs, and the SHAKE algorithm was used to constrain the bond lengths of all hydrogen-containing bonds. The trajectory files generated by the simulation were used for subsequent analysis and visualization. The above simulation process was conducted three times, and the final result represented the average of the three repetitions.

#### 2.3.5 Free energy change (ddG) prediction of wild type ATG16L1 based on AI model ThermoMPNN

To further investigate the thermal stability of wild type ATG16L1, especially the effects of amino acid mutations on protein function and stability, we used Thermodynamic Mutation Selection Neural Network (ThermoMPNN) for thermal stability prediction. ThermoMPNN is a deep learning model based on graph neural network (GNN) and transfer learning, which is specifically used to predict stability changes caused by protein point mutations (25, 26). Specifically, ThermoMPNN predicts the stability and structural changes of mutant proteins at high temperatures by inputting the amino acid sequence and mutation information of the protein, providing a reference for restoring or enhancing protein stability and function after subsequent *in vivo* simulation.

##### 2.3.5.1 Training and prediction process of the ThermoMPNN model

To efficiently run the ThermoMPNN model, we conducted model training and prediction through the Google Colab environment. We set up the running environment for ThermoMPNN in the Google Colab environment. By selecting the appropriate libraries and dependencies and loading the necessary models and datasets, we ensured smooth thermal stability prediction. Since the Colab environment provides GPU support, which is crucial for running deep learning models like ThermoMPNN that require substantial computation, it significantly accelerated the training and prediction process.

##### 2.3.5.2 PDB file upload and model execution

After setting up the ThermoMPNN runtime environment in Colab, we uploaded the PDB file of wild type ATG16L1 to the Colab system. After uploading the file, we ran the model using the default parameters in ThermoMPNN within Colab to perform thermal stability analysis of the protein after mutations. During this process, the model used the default parameters in the PyTorch framework to model the amino acid mutations, calculating the protein’s stability and melting point changes at different temperatures after the mutations.

### 2.4 Protein purification

The wild type and mutant ATG16L1 plasmids were transformed into BL21(DE3) competent cells, plated, and incubated overnight at 37 °C (Supplementary files 1, 2). Single colonies were selected and cultured in LB medium to an OD₆₀₀ of 0.6–0.8. IPTG was added to a final concentration of 0.5 mM for induction at 37 °C for 4 h, after which cells were harvested and analyzed by SDS-PAGE and Western blot. Further induction was performed with 0.2 mM and 1 mM IPTG at 37 °C and 15 °C for 4 h and 16 h, respectively. Cells were centrifuged, lysed (Tris-NaCl buffer), and sampled for expression and solubility evaluation. The optimal condition was scaled up to 2 L culture, induced at 15 °C for 16 h, followed by centrifugation, resuspension, and ultrasonication. The pellet was solubilized in denaturing buffer, centrifuged, and the supernatant was purified by Ni–NTA affinity chromatography using PBS-Urea (pH 7.4) with 50 mM and 500 mM imidazole for washing and elution, respectively. The purified product was analyzed by SDS-PAGE. Finally, the protein was refolded and concentrated in refolding buffer (PBS, 300 mM NaCl, 10% glycerol, pH 7.4) (Supplementary Figures S1, S2).

### 2.5 TSA

TSA was performed using SYPRO Orange dye. The reaction mixture, composed of 5 μL DSF buffer and 15 μL protein sample, was incubated at 25 °C for 15 min. Subsequently, 5 μL of SYPRO Orange dye was added, and the melting curve program was executed under the following conditions: 25 °C for 1 min, followed by a continuous temperature ramp to 95 °C at a rate of 0.04 °C/s with real-time fluorescence acquisition, and a final hold at 95 °C for 15 s.

### 2.6 Pull-down assay

The purified His-ATG16L1 protein (wild type or mutant) was first incubated with Ni-NTA agarose beads at 4 °C for 1 h. After washing three times with binding buffer (20 mM Tris–HCl, 100 mM NaCl, 10% glycerol, 0.1% NP-40, 10 mM imidazole, pH 8.0), the beads were mixed with pre-prepared lysate of HEK293T cells overexpressing FLAG-WIPI2b (lysis buffer: 50 mM Tris–HCl, 150 mM NaCl, 1% Triton X-100, 1 mM PMSF, protease inhibitor cocktail, pH 7.4) and incubated at 4 °C for 2 h. The beads were then washed three times with wash buffer (20 mM imidazole, other components same as binding buffer). Finally, the bound proteins were separated by SDS-PAGE and detected by Western blot using anti-FLAG antibody (1:5,000) and anti-His antibody (1:4,000).

## 3 Results

### 3.1 GWAS analysis results

#### 3.1.1 Gene annotation and pathway enrichment

**Figure 1:**
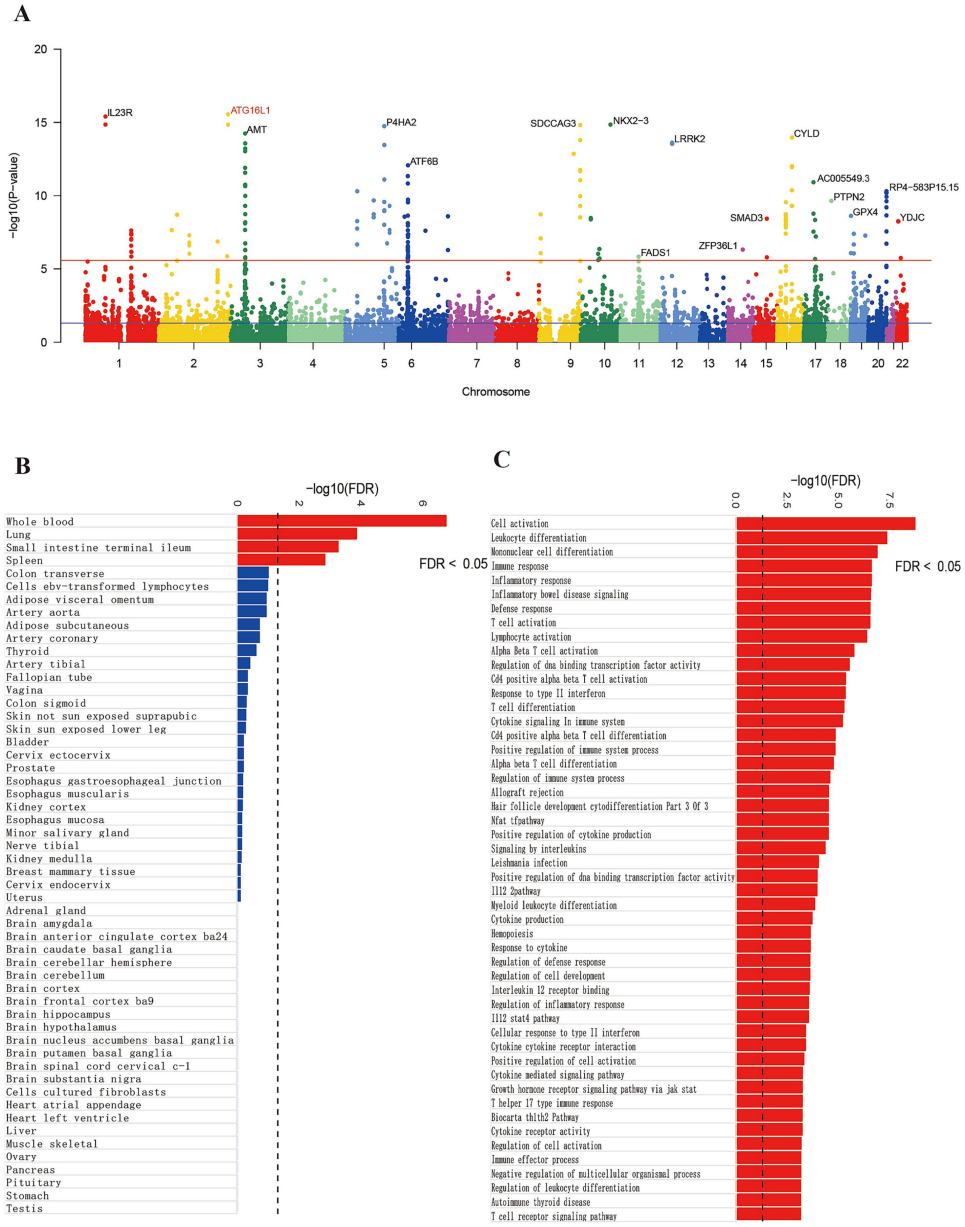
Gene annotation and pathway enrichment results. Panel **(A)** shows significant genes. Panel **(B)** shows the tissue enrichment results and panel **(C)** shows the pathway enrichment results.

Risk genes highly associated with the onset of CD were annotated using MAGMA. Following Benjamini-Hochberg correction (*p* < 0.05), 465 genes were identified as significant (Supplementary Table S2). A Manhattan plot highlighted the most prominent risk genes for each chromosome, excluding the sex chromosomes (Figure 1A). Following Benjamini-Hochberg correction for tissue-specific enrichment (Figure 1B), four tissues—whole blood, lung, terminal ileocecal part of the small intestine, and spleen—met the significance threshold (*p* < 0.05). MAGMA also identified 350 enriched gene sets (*P*_FDR_ < 0.05), with top 50 pathways linked to IBD, inflammation, and immunity (Figure 1C)—consistent with known CD mechanisms, validating MAGMA’s accuracy.

#### 3.1.2 TWAS results for CD

**Figure 2:**
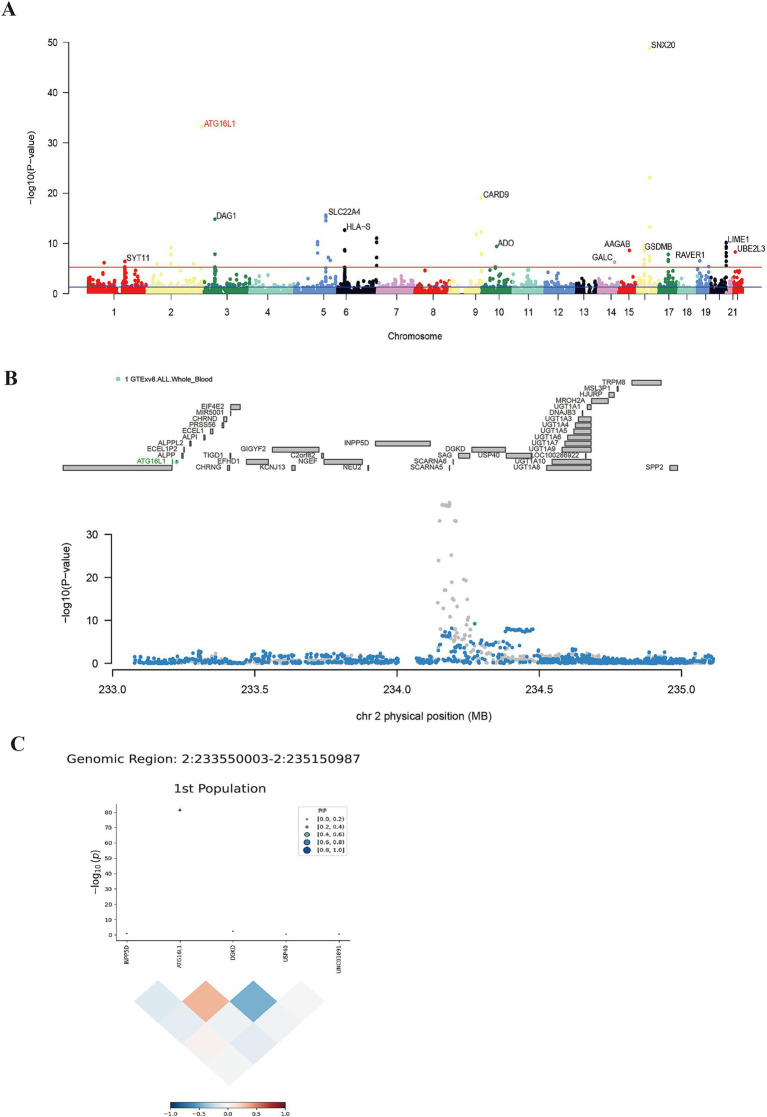
Results of single-tissue TWAS, conditional joint analysis and fine mapping. Panel **(A)** shows the positive genes screened by FUSION. The blue line indicates 5 × e^−8^, and the red line represents the threshold after FDR correction. Panel **(B)** shows the result of conditional joint analysis of ATG16L1. The top of **(B)** marks the names of genes within the region, and the gene marked in green is co-significant gene. Gray dots represent baseline TWAS signals; blue dots show post-regulation signals following green gene modulation. Panel **(C)** shows the result of ATG16L1 fine mapping, indicating that this gene plays a dominant role in this region.

Using UTMOST for cross-tissue analysis, 28 genes retained significant signals (*p* < 0.05) following Benjamini-Hochberg correction (Table 1). In single-tissue internal validation, 204 out of 8,799 genes with significant genetic expression in whole blood, as modeled in genotype data from the GTExv8 dataset, exhibited significant TWAS association signals (*p* < 0.05) following Benjamini-Hochberg correction (Supplementary Table S3). Manhattan plot showed the most prominent genes on each chromosome except the sex chromosomes (Figure 2A). In conclusion, cross-tissue and single-tissue analyses identified four overlapping candidate genes (Supplementary Table S4).

**Table 1:** The results of cross-tissue TWAS analysis of UTMOST.

Gene	Chr	Test score	*P*	*P*_FDR_
RP11-973H7.1	18	26.67	2.17E−12	8.10E-09
ATG16L1	2	331.34	2.63E−11	4.91E-08
ING5	2	15.91	3.98E−08	4.96E-05
SF3B1	2	15.15	6.44E−08	6.02E-05
PLCL1	2	13.80	1.45E−06	8.42E-04
ZFP36L2	2	12.56	1.87E−06	8.42E-04
RFTN2	2	13.62	1.14E−06	8.42E-04
PTPN2	18	12.61	2.03E−06	8.42E-04
SREBF2	22	13.01	1.73E−06	8.42E-04
USP40	2	11.92	2.45E−06	9.18E-04
SLC25A17	22	10.34	1.46E−05	4.97E-03
ROCK1	18	11.73	1.90E−05	5.56E-03
INPP5J	22	10.79	1.93E−05	5.56E-03
KREMEN1	22	10.08	2.42E−05	6.46E-03
SEH1L	18	9.84	4.00E−05	9.98E-03
FLJ31356	2	9.29	4.29E−05	1.00E-02
GTF3C3	2	8.93	7.14E−05	1.48E-02
RPL12P19	2	9.78	6.97E−05	1.48E-02
ASCC2	22	8.30	1.04E−04	2.05E-02
CCDC150	2	8.38	1.49E−04	2.53E-02
RP5-821D11.7	22	8.53	1.42E−04	2.53E-02
XRCC6	22	8.10	1.38E−04	2.53E-02
C1GALT1C1L	2	8.10	1.57E−04	2.55E-02
MKL1	22	8.52	1.94E−04	3.02E-02
RNF185	22	7.32	2.88E−04	4.30E-02
RPL9	4	6.34	3.36E−04	4.77E-02
VPREB1	22	8.68	3.45E−04	4.77E-02
ANKRD44	2	7.27	3.72E−04	4.96E-02

#### 3.1.3 Conditional and joint analysis

A conditional joint analysis was conducted to assess the conditional independence of the loci identified in this study. As shown in Table 2, there were four loci, namely the gene loci where the four genes RP11-973H7.1, PLCL1, ATG16L1, and RPL9 were located (*p* < 0.05), which represented independent signals of multiple important genes. We noticed that certain GWAS signals were influenced by gene expression that was genetically regulated. ATG16L1 predominantly contributed to the signal at the 2q37.1 locus; however, conditioning on its predicted expression notably diminishes the TWAS signal in this area (Figure 2B).

**Table 2:** Conditional and joint analyses of genes associated with CD risk.

Gene	Chr	Twas.Z	Twas.*P*	*P*_FDR_	Joint
RP11-973H7.1	18	3.32	9.13E−04	4.23E-02	TRUE
PLCL1	2	4.89	1.01E−06	1.62E-04	TRUE
ATG16L1	2	12.14	6.56E−34	2.89E-30	TRUE
RPL9	4	3.59	3.26E−04	2.02E-02	TRUE

Joint refers to whether it is a key gene for conditional joint analysis.

#### 3.1.4 The results of fine mapping

FOCUS software was employed to conduct a detailed analysis of TWAS associations using data from a European ancestry population. Under the criteria of *P*_FDR_ < 0.05 and PIP > 0.8, 30 positive genes were identified from whole blood tissue (Supplementary Table S5). FOCUS successfully created a graph depicting predicted expression correlations for each region. Figure 2C presented the TWAS summary statistics and PIP for ATG16L1.

#### 3.1.5 Results of intersection and colocalization

**Figure 3:**
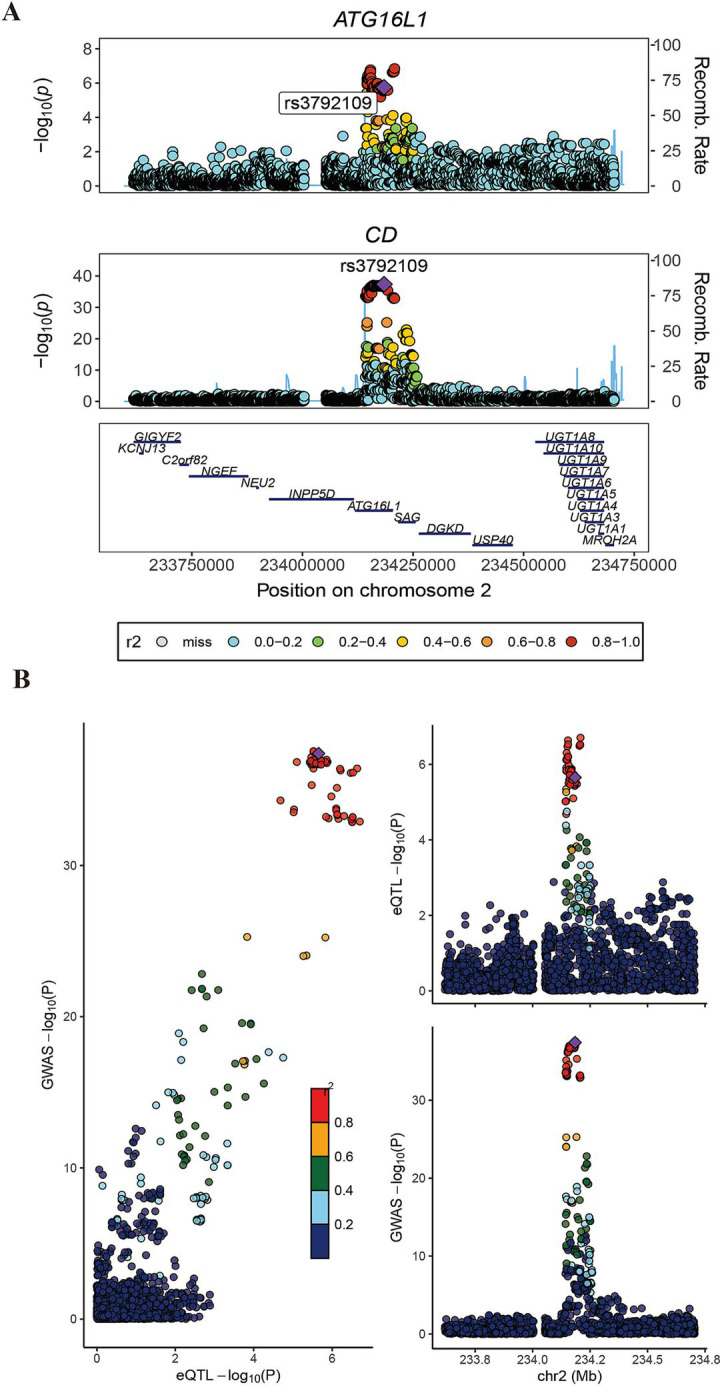
Association diagram of ATG16L1. Panels **(A,B)** show the colocalization result of ATG16L1 in the GWAS data of eQTL and CD.

Subsequently, the intersection of significant genes identified through gene annotation, cross-tissue TWAS, single-tissue TWAS, and fine mapping analysis was conducted (Supplementary Figure S3). ATG16L1 was a risk gene shared by the four methods. Subsequently, colocalization analysis was conducted. Colocalization analysis windows were configured to 500 kb, yielding a PPH4 result of 0.889 (Figure 3).

### 3.2 Results of animal experiments

#### 3.2.1 qRT-PCR

**Figure 4:**
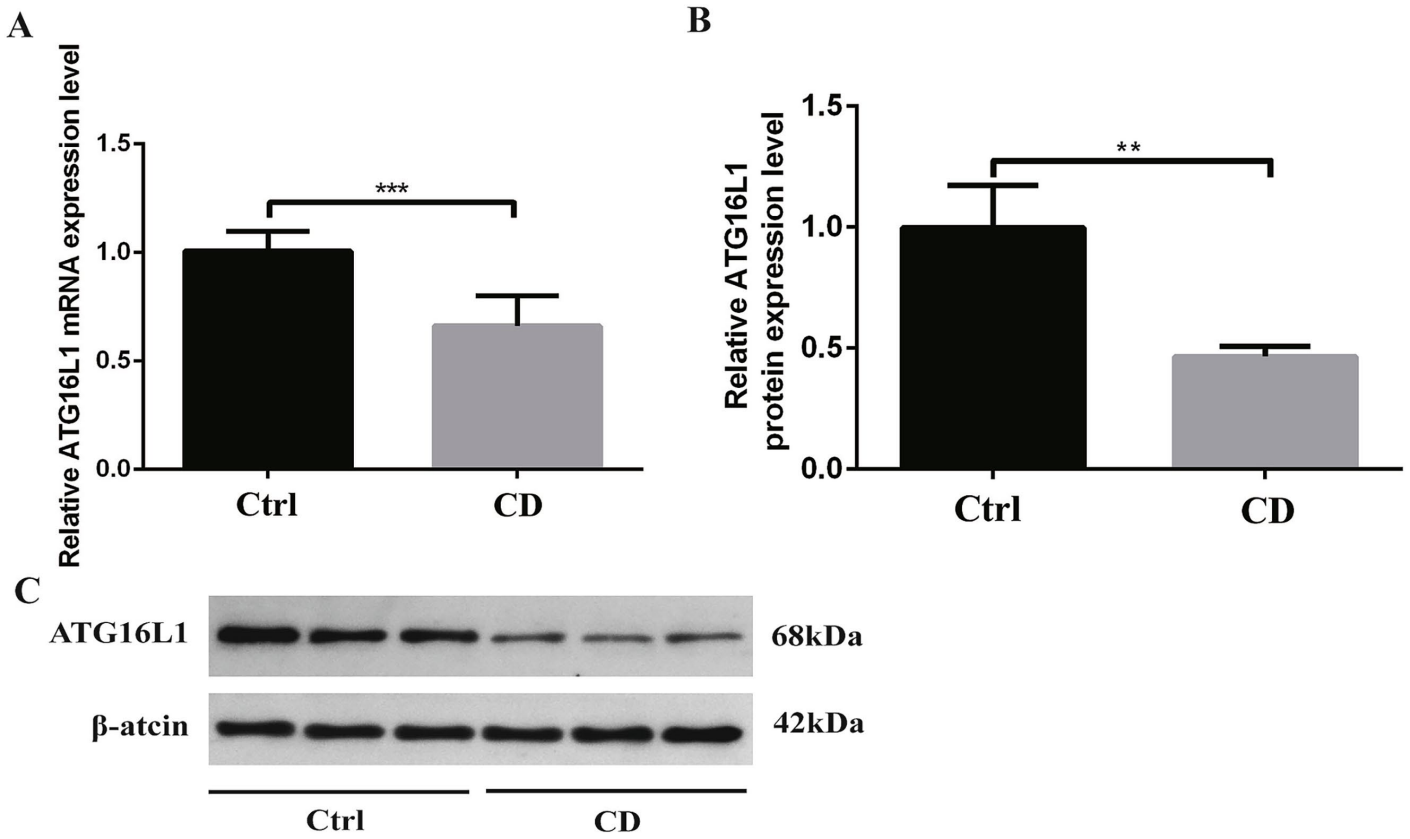
Results of qRT-PCR and Western blot. **(A)** shows mRNA expression, while **B,C** show protein expression. *p < 0.05, **p < 0.01, and ***p < 0.001.

As shown in Figure 4A, in the colon tissue of rats, the mRNA expression of ATG16L1 in CD group was lower than that in control group, and the difference was statistically significant (*p* < 0.001).

#### 3.2.2 Western blot

As shown in Figures 4B,C, in the colon tissue of rats, the expression level of ATG16L1 protein in CD group was lower than that in control group, and the difference was statistically significant (*p* < 0.01).

### 3.3 Results predicted by AI modeling

#### 3.3.1 Detailed variant information of ATG16L1 mutant rs2241880

Through investigation, we confirmed that the mutation occurs at the 300th amino acid position of ATG16L1, resulting in an amino acid substitution where the base A is replaced by G, causing the 300th threonine (T) to be replaced by alanine (A) (T300A).

#### 3.3.2 Protein structural changes after missense mutation

**Figure 5:**
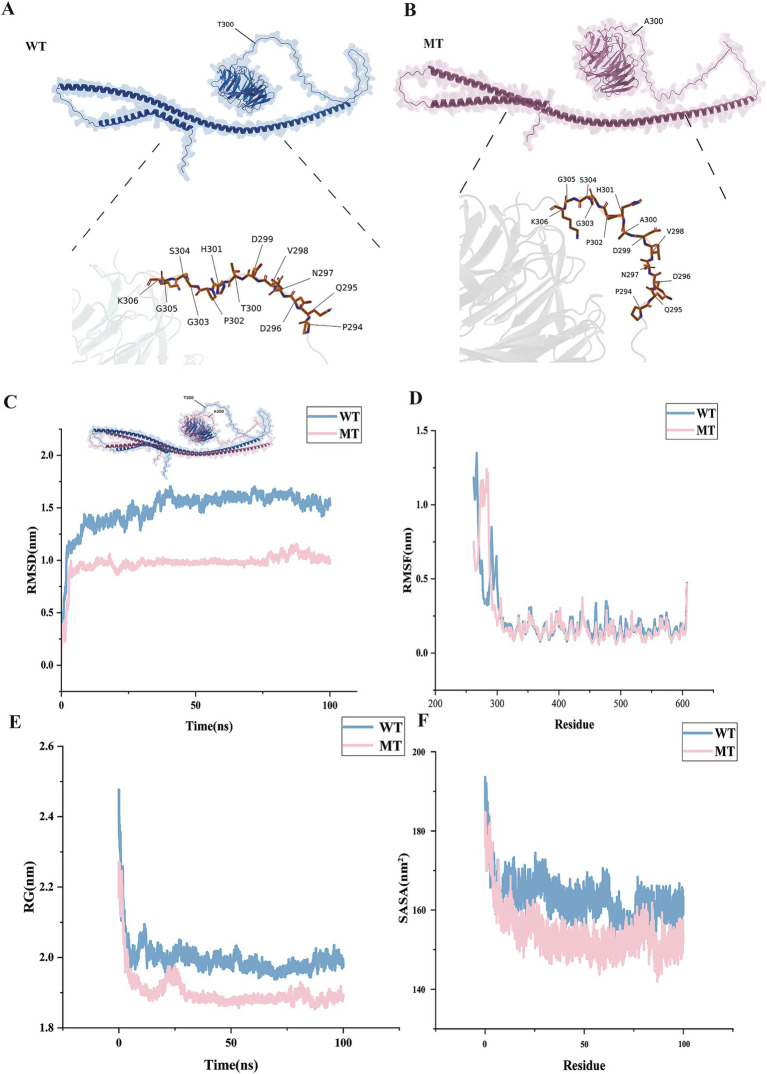
Protein structure changes and simulation results. Panels **(A,B)** show the protein structures of the wild type and mutant. Panels **(C–F)** show the molecular dynamics results. The horizontal axes represent time and amino acid position, respectively, and the vertical axis represents four molecular dynamics simulation metrics.

Figures 5A,B showed the specific morphologies of the ATG16L1 wild type and mutant proteins. It could be seen that as the 300th amino acid changed from T to A, the overall morphology of ATG16L1 underwent a certain degree of alteration. Since rs2241880 was located between the central coiled-coil domain (CCD) and WD40 domain of the protein, the morphological change in this region was more pronounced.

#### 3.3.3 Protein simulation results analysis

By simulating and analyzing the wild type and mutant ATG16L1 proteins, four simulation metrics were obtained: Root Mean Square Deviation (RMSD), Root Mean Square Fluctuation (RMSF), Radius of Gyration (RG), and Solvent Accessible Surface Area (SASA). As shown in Figure 5C, compared to the wild type, the mutant’s RMSD remained stable over time (0–100 ns), stabilizing around 1 nm, while the wild type, although also stable, maintained a value around 1.5 nm. This suggested that the mutant maintained a more stable conformation than the wild type, with enhanced protein rigidity. RMSF analysis: By comparing the RMSF values of the wild type and mutant (Figure 5D), it was found that the mutation significantly enhanced the conformational stability around the 300th amino acid. Specifically, in key functional regions (such as active sites and binding interfaces), the RMSF values of the mutant decreased, indicating increased structural stability. Mutation-induced stability changes: The RMSF fluctuation range for the mutant was reduced at several residue positions (with smaller peak-to-valley differences), suggesting that the mutation might have strengthened local hydrogen bond networks or hydrophobic interactions, thereby improving the overall structural stability. RG analysis (Figure 5E): The RG value of the mutant gradually stabilized over time (1.9 nm), while the wild type also stabilized but had a value of 2.0 nm, indicating that the mutant’s overall structure was more compact. The compact structure of the mutant might have affected the spatial arrangement of functional domains, thereby influencing the protein’s recognition and binding ability. SASA analysis (Figure 5F): The mutant’s SASA value (150–160 nm^2^) was overall lower than that of the wild type (160–170 nm^2^), indicating a reduction in the exposure of its hydrophobic core on the surface.

#### 3.3.4 Protein time-stage analysis results

**Figure 6:**
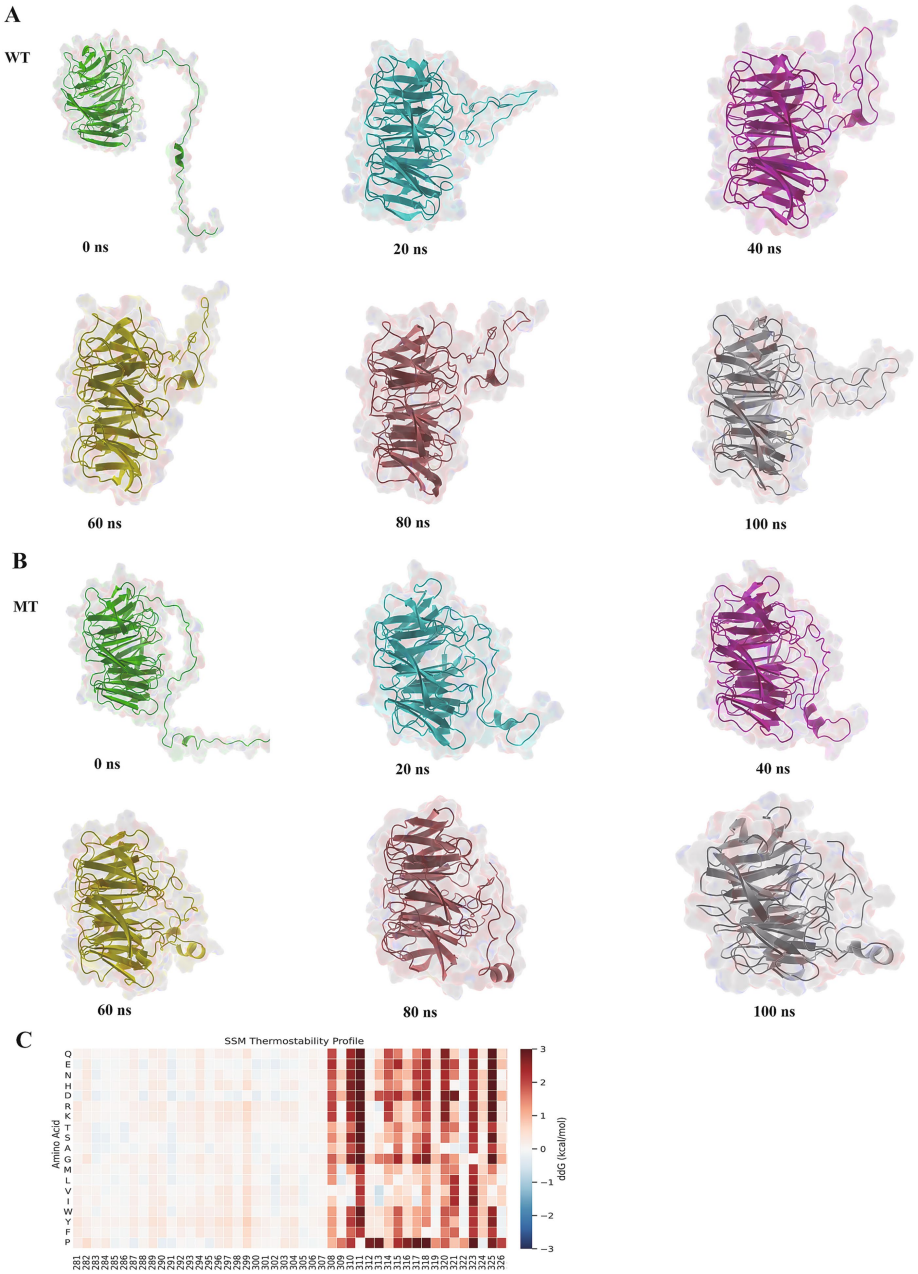
Protein time-stage analysis and ddG prediction. Panels **(A,B)** show the structural changes of the wild type and mutant during the 100 ns simulation. Panel **(C)** shows the result of the ddG prediction. In the heatmap, positive values indicate an increase in ddG, while negative values indicate a decrease in ddG. A negative value at amino acid position 300 signifies a decrease in ddG.

From the mutation results, the mutant exhibited higher structural stability: the mutant’s RMSD decreased, RG reduced, and SASA exposure was lower, indicating that its conformational flexibility was controlled and it could better maintain the protein’s rigid structure. Further analysis of the dynamic results of the protein simulation was performed by extracting representative structures from six time periods (0 to 100 ns) (Figures 6A,B) and comparing the changes between the wild type and mutant. It was found that after the mutation at the 300th amino acid, the local flexibility of the protein structure decreased, and the overall structure became more compact. As a result, the stability of the protein structure was enhanced after the mutation, which had a certain impact on the protein’s binding ability.

#### 3.3.5 Prediction of protein ddG

From the hotspot map in Figure 6C, it could be seen that after the mutation of the 300th position to A, the color changed to blue, and ddG decreased, suggesting that after the mutation, the free energy of the ATG16L1 protein was reduced, and its structural stability increased, making the ATG16L1 mutant more stable than the wild type conformation. Compared to the wild type, this might have affected its recognition and binding ability with other proteins.

### 3.4 TSA results

**Figure 7:**
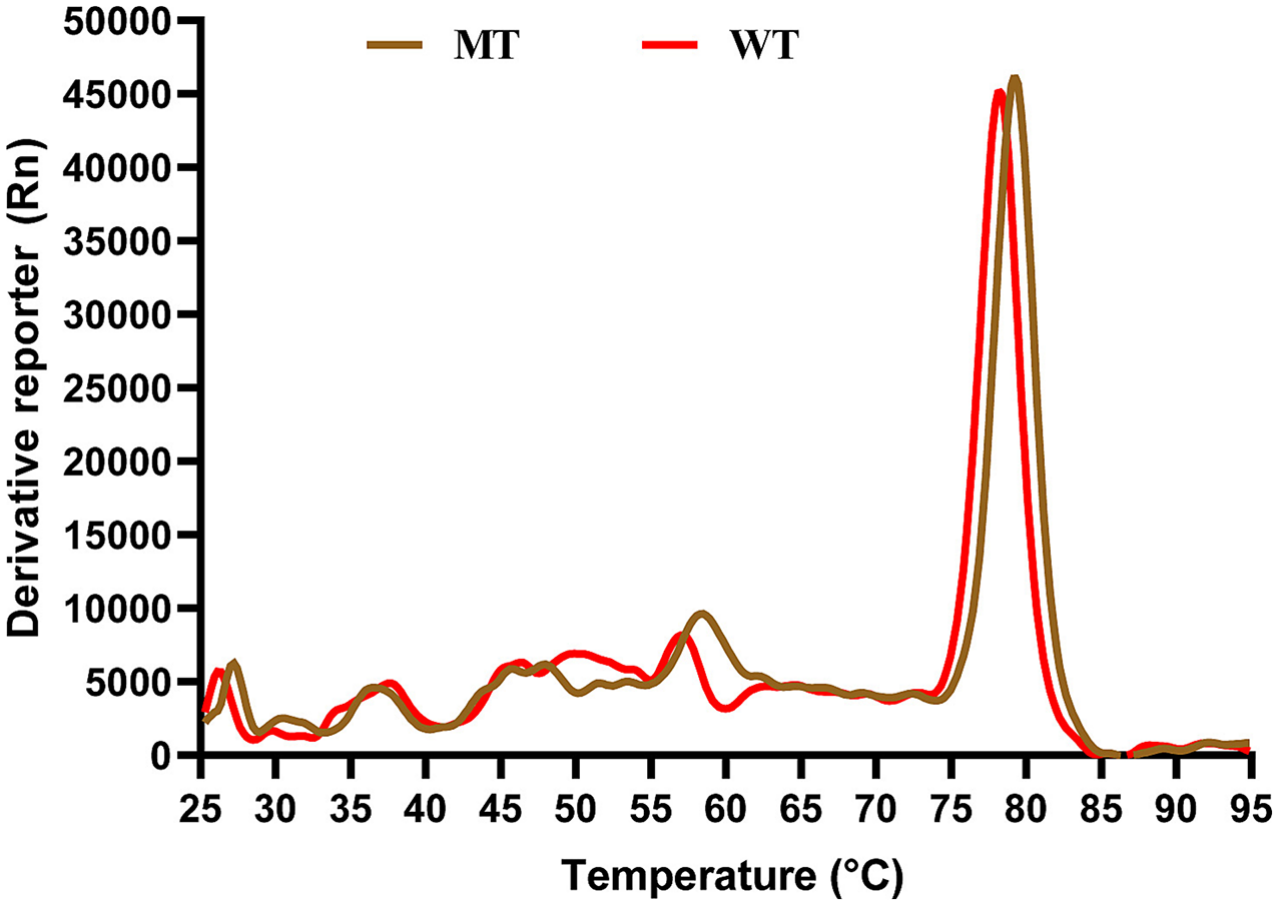
TSA results. The x-axis represents temperature, while the y-axis shows the rate of change of the fluorescence signal with temperature. The red curve corresponds to the wild type protein, and the brown curve represents the mutant protein.

Figure 7 presented the stability results of ATG16L1 wild type and mutant proteins as measured by TSA. The figure demonstrated a clear distinction in the TM values between the wild type and mutant proteins, with the mutant exhibiting a higher TM value than the wild type. A higher TM value indicated that a higher temperature was required for protein denaturation, reflecting greater protein stability. This suggested that the mutant protein possessed enhanced stability compared to the wild type.

### 3.5 Pull-down results

**Figure 8:**
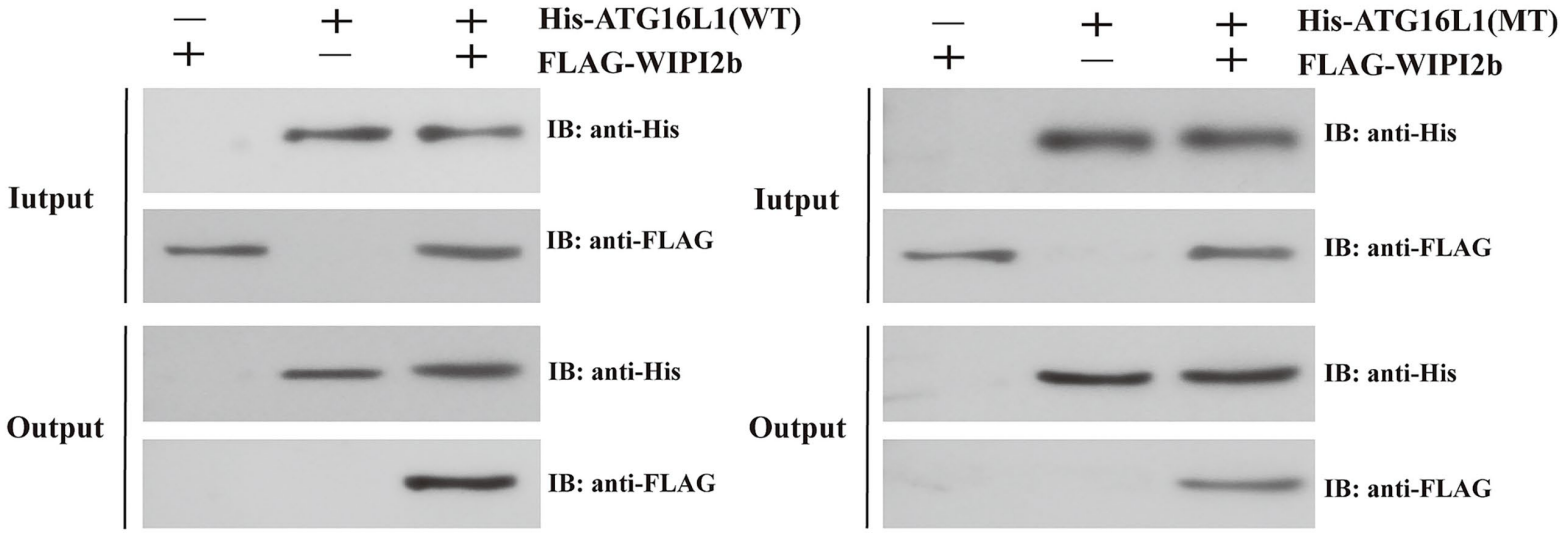
The results of the pull-down assay. The symbols “+” and “−” in the figure indicate the presence or absence of the corresponding tagged proteins, respectively. The Input group serves as the quality control, while the Output group represents the final experimental results. Focus should be placed on the anti-FLAG results in the Output group.

Figure 8 illustrated the difference in binding capacity between wild type ATG16L1 and its mutant with WIPI2b. The results from the Input group indicated that the experiment was performed successfully, as proteins with corresponding tags in each group were specifically immunoprecipitated by their respective antibodies. The results from the Output group demonstrated a significant difference in the binding affinity of the wild type and mutant proteins to WIPI2b. The amount of WIPI2b bound by the mutant was significantly lower than that bound by the wild type, providing strong supporting evidence for our computational findings.

## 4 Discussion

Based on the GWAS dataset of CD, this study systematically evaluated the genetic predictive association between gene expression and CD risk. ATG16L1 emerged as a shared gene identified by the convergence of four genetic analysis techniques: MAGMA, UTMOST, FUSION, and FOCUS. Colocalization analysis of ATG16L1 was conducted, confirming the significant impact of this signal locus on CD. Subsequently, in animal experiments, we validated the expression of ATG16L1. Compared with the control group, both mRNA and protein expression levels of ATG16L1 were decreased in the CD group. These findings can improve our understanding of the role of ATG16L1 in the genetics and pathogenesis of CD.

To further clarify the impact of protein structural changes on function, this study combined AlphaFold3 structure prediction, protein dynamics simulation, and a neural network-based thermodynamic stability prediction model to explore the impact of missense mutation at site 300 of ATG16L1 on protein structure and function. The computational predictions were validated through TSA and pull-down assays. As a protein closely related to the autophagy process, ATG16L1 has been shown by many studies to be closely related to cellular autophagy function and immune response (27–29). However, the specific mechanism of mutations on protein stability and function is still not fully understood, especially the structural changes and thermodynamic stability caused by mutations at the molecular level. This study comprehensively explained the mechanism of ATG16L1 300 site mutation from the aspects of protein stability, dynamic structural changes, and free energy changes for the first time.

ATG16L1 can be divided into three domains: the N-terminal ATG5 binding domain (ATG5BD), CCD, and WD40. Its N-terminus participates in binding to the ATG5-ATG12 complex, while its C-terminal WD40 domain mediates membrane localization and substrate recognition. ATG16L1 interacts with the WIPI2b protein through its coiled-coil domain, and WIPI2b is responsible for recruiting the ATG16L1 complex to autophagic precursor membranes, further facilitating autophagosome formation (30). The T300A mutation is located between the CCD and WD40 domains (31), adjacent to a highly conserved caspase-3 cleavage site. Several studies have shown that the T300A mutation significantly enhances caspase-3 cleavage of ATG16L1 (32). After cleavage, two fragments are produced: the N-terminal fragment contains the ATG5 binding domain but cannot localize to the autophagy initiation site due to the loss of the C-terminal region; the C-terminal fragment contains the WD40 domain but lacks ATG5 coupling ability, resulting in the loss of autophagic function (33–35).

The fact that caspase-3 significantly enhances its ability to cleave ATG16L1 after mutation should be considered from the perspective of local protein structure changes. According to the protein model predicted by modeling, the region where T300A is located does not have any secondary structure, which creates a favorable condition for caspase cleavage (36). After mutation, both the RG and SASA values were reduced, indicating increased hydrophobicity of the protein. The elevated hydrophobicity could prompt the flexible loop to collapse toward the hydrophobic core, thereby stabilizing the structure through the hydrophobic effect (37). This observation is consistent with the RMSF result, which showed enhanced structural stability near the 300th amino acid residue after mutation. Since caspase-3-mediated cleavage occurs near this position, an appropriately stable conformation may provide a more suitable binding environment for caspase-3. Additionally, the amino acid sequence from position 296 to 299 (DNVD) in ATG16L1 is adjacent to the 300th amino acid, and this sequence is similar to the DxxD sequence of caspase-3. Through the mutation, the amino acids from positions 296 to 300 change to DNVDA, which further matches the caspase-3 motif. Furthermore, the mutation leads to a significant change in the local structure at position 300, making the DNVD sequence more exposed within the cleavage range of caspase-3 compared to the wild type, thereby enhancing caspase-3’s cleavage ability (38).

Since T300A is between the CCD and WD40 domains, traditional views suggest that the mutation at this position might have a limited impact on the WD40 domain. However, evidence shows that even in the absence of caspase-3, caspase-7, and other cysteine proteases that cleave ATG16L1, T300A can still affect the functionality of the protein, which retains its full long-chain structure (39). Specifically, this mutation influences the binding ability of WD40 with common chaperone proteins, thereby affecting subsequent protein functionality. WD40 has been shown to bind to autophagy-related proteins such as WIPI2b and can also influence processes like ubiquitination and DNA damage (40). The functioning of these processes relies on the top, bottom, and circumferential surface of WD40. The formation of these three parts requires the variable region of WD40 (41). In other words, while WD40 is highly conserved in its folding, its functionality still requires some degree of variability (42). Additionally, a study analyzing the charge distribution of WD40 found that its top is predominantly hydrophobic, while the bottom is negatively charged and hydrophilic (39). Our research results indicate that after the T300A mutation, the overall structure of ATG16L1 became more compact, its flexibility decreased, and the SASA analysis showed enhanced hydrophobicity, all of which may affect the normal functioning of WD40. Furthermore, from the protein time-stage analysis results, after 100 ns simulation, the structure of the mutant showed significant differences compared to the wild type. The wild type retained the complete top, bottom and circumference of the ring structure of WD40 during the simulation. However, after 100 ns of simulation, the structure of the mutant underwent irregular changes, which may affect the functional performance of the protein. The results from the TSA and pull-down assay further support this conclusion. The TSA demonstrated increased structural stability of the mutant, while the pull-down assay revealed a significantly impaired binding affinity between the mutant and its downstream effector WIPI2b.

Interestingly, while the T300A mutation enhances structural stability, it impairs ATG16L1 function by disrupting its dimerization interface. This paradox suggests that therapeutic strategies should not aim to further stabilize the mutant protein. Instead, targeted proteolysis regulators (e.g., PROTACs) could be designed to selectively degrade the dysfunctional mutant, while allele-specific mRNA silencing or gene editing approaches could suppress its expression (43, 44). Alternatively, small molecules promoting functional dimerization without affecting stability might rescue autophagic flux in T300A carriers, offering a precision medicine avenue for CD patients with this variant.

While this study integrates computational predictions with experimental validations including TSA and pull-down assays, several limitations remain. Firstly, although TSA confirmed the altered structural stability of the ATG16L1 T300A mutant and pull-down assay revealed its impaired binding to WIPI2B, all validations were performed *in vitro*. Future studies should employ gene-editing approaches and physiological cellular models to verify these functional impacts in a more biologically relevant context. Secondly, while the ThermoMPNN model demonstrates high accuracy in thermodynamic stability prediction, there may still be limitations in predicting multi-point mutations or synergistic effects. Future research could integrate more experimental data and computational models to improve the accuracy of predictions.

## 5 Conclusion

This study elucidated the role of ATG16L1 in CD and systematically characterized the structural and functional consequences of the T300A mutation. By integrating evidence from GWAS, animal models, AlphaFold3-based structure predictions, molecular dynamics simulations, and ThermoMPNN-derived ddG calculations, we demonstrated that the T300A mutation enhanced the structural stability and hydrophobicity of ATG16L1. Experimentally, TSA confirmed the increased thermodynamic stability of the mutant, while pull-down assay revealed a significantly impaired binding capacity to WIPI2B. However, this aberrant stabilization disrupted functional dimerization and effector interactions, ultimately compromising protein function. These findings provide mechanistic insights into ATG16L1 dysfunction in CD and offer a theoretical basis for future therapeutic strategies targeting this mutation.

## Data Availability

The original contributions presented in the study are included in the article/Supplementary material, further inquiries can be directed to the corresponding authors.

## Footnotes

^1^ https://www.ncbi.nlm.nih.gov/

^2^ https://www.ensembl.org/

^3^ https://www.genecards.org/

^4^ https://www.uniprot.org/

^5^ https://alphafoldserver.com/
