# Supplementary material for: The role of ATG16L1 in Crohn’s disease and the structural alteration mechanisms and functional consequences of the rs2241880 variant
Source: Front Med (Lausanne). 2025 Oct 3;12:1656575. doi: 10.3389/fmed.2025.1656575 (PMC12531253; doi:10.3389/fmed.2025.1656575)

**Table S1 Protein sequence of wild type ATG16L1**

| **Protein** | **Sequence** |
| --- | --- |
| ATG16L1 | MSSGLRAADFPRWKRHISEQLRRRDRLQRQAFEEIILQYNKLLEKSDLHSVLAQKLQAEKHDVPNRHEISPGHDGTWNDNQLQEMAQLRIKHQEELTELHKKRGELAQLVIDLNNQMQRKDREMQMNEAKIAECLQTISDLETECLDLRTKLCDLERANQTLKDEYDALQITFTALEGKLRKTTEENQELVTRWMAEKAQEANRLNAENEKDSRRRQARLQKELAEAAKEPLPVEQDDDIEVIVDETSDHTEETSPVRAISRAATKRLSQPAGGLLDSITNIFGRRSVSSFPVPQDNVDTHPGSGKEVRVPATALCVFDAHDGEVNAVQFSPGSRLLATGGMDRRVKLWEVFGEKCEFKGSLSGSNAGITSIEFDSAGSYLLAASNDFASRIWTVDDYRLRHTLTGHSGKVLSAKFLLDNARIVSGSHDRTLKLWDLRSKVCIKTVFAGSSCNDIVCTEQCVMSGHFDKKIRFWDIRSESIVREMELLGKITALDLNPERTELLSCSRDDLLKVIDLRTNAIKQTFSAPGFKCGSDWTRVVFSPDGSYVAAGSAEGSLYIWSVLTGKVEKVLSKQHSSSINAVAWSPSGSHVVSVDKGCKAVLWAQY |

**Table S2 CD risk genes identified through gene-based association studies**

| **GENE** | **CHR** | **N** | **ZSTAT** | ***P*** | ***P*_FDR_** |
| --- | --- | --- | --- | --- | --- |
| ATG16L1 | 2 | 20883 | 8.0984 | 2.78E-16 | 3.80E-12 |
| IL23R | 1 | 20883 | 8.0544 | 3.99E-16 | 3.80E-12 |
| C1orf141 | 1 | 20883 | 7.8989 | 1.41E-15 | 4.86E-12 |
| NKX2-3 | 10 | 20883 | 7.8985 | 1.41E-15 | 4.86E-12 |
| SAG | 2 | 20883 | 7.8957 | 1.44E-15 | 4.86E-12 |
| SDCCAG3 | 9 | 20883 | 7.8883 | 1.53E-15 | 4.86E-12 |
| P4HA2 | 5 | 20883 | 7.8679 | 1.80E-15 | 4.90E-12 |
| AMT | 3 | 20883 | 7.7228 | 5.69E-15 | 1.35E-11 |
| CYLD | 16 | 20883 | 7.642 | 1.07E-14 | 2.26E-11 |
| SNAPC4 | 9 | 20883 | 7.588 | 1.62E-14 | 3.09E-11 |
| LRRK2 | 12 | 20883 | 7.5352 | 2.44E-14 | 4.22E-11 |
| APEH | 3 | 20883 | 7.5218 | 2.70E-14 | 4.22E-11 |
| MUC19 | 12 | 20883 | 7.5132 | 2.88E-14 | 4.22E-11 |
| C5orf56 | 5 | 20883 | 7.4884 | 3.49E-14 | 4.73E-11 |
| MST1 | 3 | 20883 | 7.4132 | 6.16E-14 | 7.81E-11 |
| NICN1 | 3 | 20883 | 7.3593 | 9.24E-14 | 1.10E-10 |
| TNFSF15 | 9 | 20883 | 7.3034 | 1.40E-13 | 1.57E-10 |
| ATF6B | 6 | 20883 | 7.0567 | 8.52E-13 | 9.01E-10 |
| SNX20 | 16 | 20883 | 7.0373 | 9.80E-13 | 9.81E-10 |
| NKD1 | 16 | 20883 | 7.0158 | 1.14E-12 | 1.09E-09 |
| TCTA | 3 | 20883 | 7.0012 | 1.27E-12 | 1.15E-09 |
| INPP5E | 9 | 20883 | 6.9514 | 1.81E-12 | 1.56E-09 |
| PMPCA | 9 | 20883 | 6.9275 | 2.14E-12 | 1.77E-09 |
| BSN | 3 | 20883 | 6.8933 | 2.73E-12 | 2.16E-09 |
| LST1 | 6 | 20883 | 6.8183 | 4.61E-12 | 3.50E-09 |
| SLC22A5 | 5 | 20883 | 6.7394 | 7.95E-12 | 5.82E-09 |
| C9orf163 | 9 | 20883 | 6.7238 | 8.85E-12 | 6.24E-09 |
| AC005549.3 | 17 | 20883 | 6.6785 | 1.21E-11 | 8.20E-09 |
| C6orf47 | 6 | 20883 | 6.6494 | 1.47E-11 | 9.65E-09 |
| RNF123 | 3 | 20883 | 6.6199 | 1.80E-11 | 1.14E-08 |
| RHOA | 3 | 20883 | 6.6007 | 2.05E-11 | 1.26E-08 |
| CDHR4 | 3 | 20883 | 6.5868 | 2.25E-11 | 1.34E-08 |
| BRD7 | 16 | 20883 | 6.4899 | 4.29E-11 | 2.47E-08 |
| RPL37 | 5 | 20883 | 6.4669 | 5.00E-11 | 2.72E-08 |
| RP4-583P15.15 | 20 | 20883 | 6.4666 | 5.01E-11 | 2.72E-08 |
| ZGPAT | 20 | 20883 | 6.4394 | 6.00E-11 | 3.17E-08 |
| ARFRP1 | 20 | 20883 | 6.4345 | 6.19E-11 | 3.18E-08 |
| TNFRSF6B | 20 | 20883 | 6.4258 | 6.56E-11 | 3.28E-08 |
| TRAIP | 3 | 20883 | 6.3552 | 1.04E-10 | 5.08E-08 |
| SEC16A | 9 | 20883 | 6.3482 | 1.09E-10 | 5.18E-08 |
| SLC2A4RG | 20 | 20883 | 6.3342 | 1.19E-10 | 5.53E-08 |
| TNXB | 6 | 20883 | 6.2631 | 1.89E-10 | 8.54E-08 |
| LNPEP | 5 | 20883 | 6.2452 | 2.12E-10 | 9.36E-08 |
| PTPN2 | 18 | 20883 | 6.2344 | 2.27E-10 | 9.80E-08 |
| LIME1 | 20 | 20883 | 6.2263 | 2.39E-10 | 1.01E-07 |
| PDLIM4 | 5 | 20883 | 6.2075 | 2.69E-10 | 1.11E-07 |
| NPIPB8 | 16 | 20883 | 6.2011 | 2.80E-10 | 1.12E-07 |
| STK19 | 6 | 20883 | 6.1997 | 2.83E-10 | 1.12E-07 |
| ADCY7 | 16 | 20883 | 6.1094 | 5.00E-10 | 1.79E-07 |
| NOD2 | 16 | 20883 | 6.1094 | 5.00E-10 | 1.79E-07 |
| DAG1 | 3 | 20883 | 6.1094 | 5.00E-10 | 1.79E-07 |
| CARD9 | 9 | 20883 | 6.1094 | 5.00E-10 | 1.79E-07 |
| IRGM | 5 | 20883 | 6.1094 | 5.00E-10 | 1.79E-07 |
| RTEL1-TNFRSF6B | 20 | 20883 | 6.0721 | 6.31E-10 | 2.22E-07 |
| IRF1 | 5 | 20883 | 5.9979 | 1.00E-09 | 3.46E-07 |
| SLC22A4 | 5 | 20883 | 5.9821 | 1.10E-09 | 3.74E-07 |
| CCL2 | 17 | 20883 | 5.9115 | 1.70E-09 | 5.66E-07 |
| IP6K1 | 3 | 20883 | 5.8985 | 1.83E-09 | 5.90E-07 |
| ERAP2 | 5 | 20883 | 5.8963 | 1.86E-09 | 5.90E-07 |
| CLN3 | 16 | 20883 | 5.8957 | 1.87E-09 | 5.90E-07 |
| JAK2 | 9 | 20883 | 5.8923 | 1.90E-09 | 5.90E-07 |
| SULT1A1 | 16 | 20883 | 5.8897 | 1.93E-09 | 5.90E-07 |
| CLN3 | 16 | 20883 | 5.888 | 1.95E-09 | 5.90E-07 |
| PUS10 | 2 | 20883 | 5.8842 | 2.00E-09 | 5.94E-07 |
| RTEL1 | 20 | 20883 | 5.8671 | 2.22E-09 | 6.49E-07 |
| GPANK1 | 6 | 20883 | 5.8609 | 2.30E-09 | 6.64E-07 |
| GPX4 | 19 | 20883 | 5.8547 | 2.39E-09 | 6.78E-07 |
| RNASET2 | 6 | 20883 | 5.843 | 2.56E-09 | 7.17E-07 |
| CDKAL1 | 6 | 20883 | 5.8335 | 2.71E-09 | 7.48E-07 |
| CTD-2260A17.2 | 5 | 20883 | 5.8173 | 2.99E-09 | 8.02E-07 |
| DNLZ | 9 | 20883 | 5.8153 | 3.03E-09 | 8.02E-07 |
| SH2B1 | 16 | 20883 | 5.8148 | 3.04E-09 | 8.02E-07 |
| CUL2 | 10 | 20883 | 5.7982 | 3.35E-09 | 8.73E-07 |
| SMAD3 | 15 | 20883 | 5.7801 | 3.73E-09 | 9.59E-07 |
| CREM | 10 | 20883 | 5.7636 | 4.12E-09 | 1.04E-06 |
| TUFM | 16 | 20883 | 5.7624 | 4.15E-09 | 1.04E-06 |
| ZPBP2 | 17 | 20883 | 5.7467 | 4.55E-09 | 1.11E-06 |
| APOBR | 16 | 20883 | 5.7462 | 4.56E-09 | 1.11E-06 |
| ATXN2L | 16 | 20883 | 5.7182 | 5.38E-09 | 1.30E-06 |
| PRKAA1 | 5 | 20883 | 5.7147 | 5.49E-09 | 1.30E-06 |
| YDJC | 22 | 20883 | 5.7137 | 5.53E-09 | 1.30E-06 |
| UBE2L3 | 22 | 20883 | 5.7051 | 5.81E-09 | 1.35E-06 |
| UBA7 | 3 | 20883 | 5.6841 | 6.57E-09 | 1.51E-06 |
| NFKBIL1 | 6 | 20883 | 5.6596 | 7.58E-09 | 1.72E-06 |
| GPX1 | 3 | 20883 | 5.6557 | 7.76E-09 | 1.74E-06 |
| SULT1A2 | 16 | 20883 | 5.6407 | 8.47E-09 | 1.87E-06 |
| CSF2 | 5 | 20883 | 5.6153 | 9.81E-09 | 2.14E-06 |
| CCDC101 | 16 | 20883 | 5.6092 | 1.02E-08 | 2.20E-06 |
| CLIC1 | 6 | 20883 | 5.5967 | 1.09E-08 | 2.33E-06 |
| ATP2A1 | 16 | 20883 | 5.5416 | 1.50E-08 | 3.17E-06 |
| TTC33 | 5 | 20883 | 5.5141 | 1.75E-08 | 3.66E-06 |
| USP4 | 3 | 20883 | 5.4743 | 2.20E-08 | 4.50E-06 |
| AC010441.1 | 5 | 20883 | 5.4741 | 2.20E-08 | 4.50E-06 |
| THADA | 2 | 20883 | 5.4691 | 2.26E-08 | 4.58E-06 |
| XXbac-BPG32J3.20 | 6 | 20883 | 5.4645 | 2.32E-08 | 4.65E-06 |
| ASH1L | 1 | 20883 | 5.4588 | 2.40E-08 | 4.75E-06 |
| BACH2 | 6 | 20883 | 5.4534 | 2.47E-08 | 4.85E-06 |
| RP4-583P15.14 | 20 | 20883 | 5.4336 | 2.76E-08 | 5.36E-06 |
| CCL11 | 17 | 20883 | 5.4268 | 2.87E-08 | 5.51E-06 |
| SMIM3 | 5 | 20883 | 5.3964 | 3.40E-08 | 6.47E-06 |
| HCN3 | 1 | 20883 | 5.3767 | 3.79E-08 | 7.14E-06 |
| IL27 | 16 | 20883 | 5.3721 | 3.89E-08 | 7.25E-06 |
| S1PR5 | 19 | 20883 | 5.367 | 4.00E-08 | 7.39E-06 |
| FDPS | 1 | 20883 | 5.3618 | 4.12E-08 | 7.53E-06 |
| HLA-DOB | 6 | 20883 | 5.3538 | 4.31E-08 | 7.80E-06 |
| SCAMP3 | 1 | 20883 | 5.3446 | 4.53E-08 | 8.13E-06 |
| ATP6V1G2-DDX39B | 6 | 20883 | 5.3303 | 4.90E-08 | 8.71E-06 |
| IL18RAP | 2 | 20883 | 5.3235 | 5.09E-08 | 8.97E-06 |
| FUT2 | 19 | 20883 | 5.3146 | 5.34E-08 | 9.32E-06 |
| STAT3 | 17 | 20883 | 5.2888 | 6.16E-08 | 1.06E-05 |
| NELFE | 6 | 20883 | 5.278 | 6.53E-08 | 1.12E-05 |
| INSL6 | 9 | 20883 | 5.2343 | 8.28E-08 | 1.41E-05 |
| PKLR | 1 | 20883 | 5.2183 | 9.03E-08 | 1.52E-05 |
| CLK2 | 1 | 20883 | 5.1989 | 1.00E-07 | 1.67E-05 |
| PLCL1 | 2 | 20883 | 5.1425 | 1.36E-07 | 2.24E-05 |
| VWA7 | 6 | 20883 | 5.1255 | 1.48E-07 | 2.43E-05 |
| IL1RL1 | 2 | 20883 | 5.1135 | 1.58E-07 | 2.57E-05 |
| NDFIP1 | 5 | 20883 | 5.0904 | 1.79E-07 | 2.88E-05 |
| CAMKV | 3 | 20883 | 5.0872 | 1.82E-07 | 2.90E-05 |
| STMN3 | 20 | 20883 | 5.0791 | 1.90E-07 | 3.01E-05 |
| PTGER4 | 5 | 20883 | 5.0569 | 2.13E-07 | 3.35E-05 |
| CDC37 | 19 | 20883 | 5.0553 | 2.15E-07 | 3.35E-05 |
| IL18R1 | 2 | 20883 | 5.0264 | 2.50E-07 | 3.86E-05 |
| FAM189B | 1 | 20883 | 5.0184 | 2.60E-07 | 4.00E-05 |
| VARS | 6 | 20883 | 5.0098 | 2.72E-07 | 4.14E-05 |
| MICB | 6 | 20883 | 4.9592 | 3.54E-07 | 5.34E-05 |
| TNF | 6 | 20883 | 4.9566 | 3.59E-07 | 5.37E-05 |
| ZNF365 | 10 | 20883 | 4.9187 | 4.36E-07 | 6.47E-05 |
| ZFP36L1 | 14 | 20883 | 4.8989 | 4.82E-07 | 7.10E-05 |
| HLA-B | 6 | 20883 | 4.8862 | 5.14E-07 | 7.36E-05 |
| GMPPB | 3 | 20883 | 4.8861 | 5.14E-07 | 7.36E-05 |
| AMIGO3 | 3 | 20883 | 4.8861 | 5.14E-07 | 7.36E-05 |
| FGFR1OP | 6 | 20883 | 4.886 | 5.15E-07 | 7.36E-05 |
| MSTO1 | 1 | 20883 | 4.8322 | 6.75E-07 | 9.58E-05 |
| RIT1 | 1 | 20883 | 4.8249 | 7.00E-07 | 9.87E-05 |
| C2 | 6 | 20883 | 4.8205 | 7.16E-07 | 1.00E-04 |
| SBNO2 | 19 | 20883 | 4.7908 | 8.31E-07 | 1.15E-04 |
| INSL4 | 9 | 20883 | 4.7896 | 8.36E-07 | 1.15E-04 |
| PDE4A | 19 | 20883 | 4.7814 | 8.70E-07 | 1.19E-04 |
| SLC9A4 | 2 | 20883 | 4.7712 | 9.16E-07 | 1.24E-04 |
| IPMK | 10 | 20883 | 4.7671 | 9.35E-07 | 1.26E-04 |
| CYP21A2 | 6 | 20883 | 4.7234 | 1.16E-06 | 1.55E-04 |
| TAP2 | 6 | 20883 | 4.708 | 1.25E-06 | 1.66E-04 |
| SP140 | 2 | 20883 | 4.6886 | 1.38E-06 | 1.82E-04 |
| YY1AP1 | 1 | 20883 | 4.6828 | 1.41E-06 | 1.86E-04 |
| FADS1 | 11 | 20883 | 4.6756 | 1.47E-06 | 1.91E-04 |
| CFB | 6 | 20883 | 4.6587 | 1.59E-06 | 2.06E-04 |
| RP11-342M21.2 | 15 | 20883 | 4.6552 | 1.62E-06 | 2.07E-04 |
| MON1A | 3 | 20883 | 4.6547 | 1.62E-06 | 2.07E-04 |
| MSH5 | 6 | 20883 | 4.6518 | 1.65E-06 | 2.09E-04 |
| CFB | 6 | 20883 | 4.6477 | 1.68E-06 | 2.11E-04 |
| HORMAD2 | 22 | 20883 | 4.6333 | 1.80E-06 | 2.25E-04 |
| MST1R | 3 | 20883 | 4.6283 | 1.84E-06 | 2.29E-04 |
| EGR2 | 10 | 20883 | 4.6181 | 1.94E-06 | 2.39E-04 |
| ORMDL3 | 17 | 20883 | 4.5992 | 2.12E-06 | 2.60E-04 |
| MSH5-SAPCD1 | 6 | 20883 | 4.5969 | 2.14E-06 | 2.61E-04 |
| ADO | 10 | 20883 | 4.593 | 2.18E-06 | 2.65E-04 |
| PRRC2A | 6 | 20883 | 4.5901 | 2.22E-06 | 2.67E-04 |
| CISD1 | 10 | 20883 | 4.5631 | 2.52E-06 | 3.01E-04 |
| NCR3 | 6 | 20883 | 4.549 | 2.69E-06 | 3.20E-04 |
| REL | 2 | 20883 | 4.5436 | 2.76E-06 | 3.27E-04 |
| NOTCH1 | 9 | 20883 | 4.538 | 2.84E-06 | 3.33E-04 |
| PSORS1C1 | 6 | 20883 | 4.527 | 2.99E-06 | 3.48E-04 |
| FADS2 | 11 | 20883 | 4.5264 | 3.00E-06 | 3.48E-04 |
| AL161450.1 | 9 | 20883 | 4.5199 | 3.09E-06 | 3.57E-04 |
| PER3 | 1 | 20883 | 4.5169 | 3.14E-06 | 3.60E-04 |
| RBM5 | 3 | 20883 | 4.4536 | 4.22E-06 | 4.81E-04 |
| IP6K2 | 3 | 20883 | 4.4214 | 4.90E-06 | 5.55E-04 |
| DNMT3A | 2 | 20883 | 4.3923 | 5.61E-06 | 6.31E-04 |
| IMPDH2 | 3 | 20883 | 4.3888 | 5.70E-06 | 6.38E-04 |
| GMEB2 | 20 | 20883 | 4.3712 | 6.18E-06 | 6.87E-04 |
| RABEP2 | 16 | 20883 | 4.3593 | 6.52E-06 | 7.21E-04 |
| STAT5A | 17 | 20883 | 4.3392 | 7.15E-06 | 7.86E-04 |
| PTRF | 17 | 20883 | 4.3337 | 7.33E-06 | 8.01E-04 |
| MIER1 | 1 | 20883 | 4.3322 | 7.38E-06 | 8.01E-04 |
| HELZ2 | 20 | 20883 | 4.3312 | 7.42E-06 | 8.01E-04 |
| TAP2 | 6 | 20883 | 4.324 | 7.66E-06 | 8.23E-04 |
| MICA | 6 | 20883 | 4.3201 | 7.80E-06 | 8.33E-04 |
| MAP3K8 | 10 | 20883 | 4.3046 | 8.36E-06 | 8.89E-04 |
| GBA | 1 | 20883 | 4.3026 | 8.44E-06 | 8.92E-04 |
| IL12B | 5 | 20883 | 4.2878 | 9.02E-06 | 9.48E-04 |
| GSDMB | 17 | 20883 | 4.278 | 9.43E-06 | 9.86E-04 |
| RBM6 | 3 | 20883 | 4.2761 | 9.51E-06 | 9.88E-04 |
| TMEM258 | 11 | 20883 | 4.2657 | 9.96E-06 | 1.03E-03 |
| MIEN1 | 17 | 20883 | 4.2635 | 1.01E-05 | 1.03E-03 |
| RFT1 | 3 | 20883 | 4.2456 | 1.09E-05 | 1.11E-03 |
| IL12RB2 | 1 | 20883 | 4.2411 | 1.11E-05 | 1.13E-03 |
| STAT5B | 17 | 20883 | 4.234 | 1.15E-05 | 1.16E-03 |
| CCDC88B | 11 | 20883 | 4.2332 | 1.15E-05 | 1.16E-03 |
| PLCB3 | 11 | 20883 | 4.2144 | 1.25E-05 | 1.25E-03 |
| BOLL | 2 | 20883 | 4.2133 | 1.26E-05 | 1.25E-03 |
| CCDC36 | 3 | 20883 | 4.2065 | 1.30E-05 | 1.28E-03 |
| CD19 | 16 | 20883 | 4.201 | 1.33E-05 | 1.31E-03 |
| UBLCP1 | 5 | 20883 | 4.1973 | 1.35E-05 | 1.32E-03 |
| CTD-2330K9.3 | 3 | 20883 | 4.1966 | 1.35E-05 | 1.32E-03 |
| CELSR3 | 3 | 20883 | 4.1875 | 1.41E-05 | 1.37E-03 |
| SOCS1 | 16 | 20883 | 4.1665 | 1.55E-05 | 1.49E-03 |
| ERBB2 | 17 | 20883 | 4.1662 | 1.55E-05 | 1.49E-03 |
| DDX39B | 6 | 20883 | 4.1576 | 1.61E-05 | 1.54E-03 |
| QARS | 3 | 20883 | 4.1488 | 1.67E-05 | 1.59E-03 |
| TNFRSF4 | 1 | 20883 | 4.1451 | 1.70E-05 | 1.61E-03 |
| KLHDC8B | 3 | 20883 | 4.1432 | 1.71E-05 | 1.61E-03 |
| CTD-2369P2.10 | 19 | 20883 | 4.1208 | 1.89E-05 | 1.76E-03 |
| FDX1L | 19 | 20883 | 4.1208 | 1.89E-05 | 1.76E-03 |
| NCKIPSD | 3 | 20883 | 4.1124 | 1.96E-05 | 1.82E-03 |
| RP11-595B24.2 | 18 | 20883 | 4.11 | 1.98E-05 | 1.82E-03 |
| SMIM19 | 8 | 20883 | 4.1092 | 1.99E-05 | 1.82E-03 |
| C3orf62 | 3 | 20883 | 4.1027 | 2.04E-05 | 1.87E-03 |
| AC011997.1 | 2 | 20883 | 4.0903 | 2.15E-05 | 1.96E-03 |
| ZFP36L2 | 2 | 20883 | 4.0756 | 2.30E-05 | 2.08E-03 |
| OTUD7A | 15 | 20883 | 4.0698 | 2.35E-05 | 2.12E-03 |
| CSNK2B-LY6G5B-1181 | 6 | 20883 | 4.0583 | 2.47E-05 | 2.22E-03 |
| AKAP11 | 13 | 20883 | 4.0519 | 2.54E-05 | 2.27E-03 |
| VARS2 | 6 | 20883 | 4.0399 | 2.67E-05 | 2.38E-03 |
| C1orf106 | 1 | 20883 | 4.0369 | 2.71E-05 | 2.40E-03 |
| RPS6KB1 | 17 | 20883 | 4.0277 | 2.82E-05 | 2.48E-03 |
| THBS3 | 1 | 20883 | 4.0256 | 2.84E-05 | 2.49E-03 |
| MTMR3 | 22 | 20883 | 4.0227 | 2.88E-05 | 2.51E-03 |
| EHMT2 | 6 | 20883 | 4.0195 | 2.92E-05 | 2.53E-03 |
| RFTN2 | 2 | 20883 | 4.0139 | 2.99E-05 | 2.58E-03 |
| ATP6V1G2 | 6 | 20883 | 4.0133 | 2.99E-05 | 2.58E-03 |
| SSR2 | 1 | 20883 | 4.0095 | 3.04E-05 | 2.61E-03 |
| FERMT1 | 20 | 20883 | 4.008 | 3.06E-05 | 2.61E-03 |
| SLC2A13 | 12 | 20883 | 4.0075 | 3.07E-05 | 2.61E-03 |
| USP1 | 1 | 20883 | 4.003 | 3.13E-05 | 2.64E-03 |
| GSDMA | 17 | 20883 | 3.9998 | 3.17E-05 | 2.67E-03 |
| ZMIZ1 | 10 | 20883 | 3.9843 | 3.38E-05 | 2.83E-03 |
| RPS6KA4 | 11 | 20883 | 3.9833 | 3.40E-05 | 2.83E-03 |
| AL138847.1 | 1 | 20883 | 3.9746 | 3.53E-05 | 2.93E-03 |
| CDKN2D | 19 | 20883 | 3.9701 | 3.59E-05 | 2.97E-03 |
| ZNF300 | 5 | 20883 | 3.9668 | 3.64E-05 | 3.00E-03 |
| MTX1 | 1 | 20883 | 3.9549 | 3.83E-05 | 3.14E-03 |
| GPR183 | 13 | 20883 | 3.9485 | 3.93E-05 | 3.21E-03 |
| HLA-DOA | 6 | 20883 | 3.9469 | 3.96E-05 | 3.22E-03 |
| LTBR | 12 | 20883 | 3.9377 | 4.11E-05 | 3.33E-03 |
| RSPO3 | 6 | 20883 | 3.9056 | 4.70E-05 | 3.79E-03 |
| MOB4 | 2 | 20883 | 3.9002 | 4.81E-05 | 3.85E-03 |
| SLC20A2 | 8 | 20883 | 3.8997 | 4.81E-05 | 3.85E-03 |
| CCDC122 | 13 | 20883 | 3.8935 | 4.94E-05 | 3.91E-03 |
| TUBD1 | 17 | 20883 | 3.8932 | 4.95E-05 | 3.91E-03 |
| AP4B1 | 1 | 20883 | 3.8926 | 4.96E-05 | 3.91E-03 |
| DOCK7 | 1 | 20883 | 3.8699 | 5.44E-05 | 4.27E-03 |
| HLA-DQB2 | 6 | 20883 | 3.8697 | 5.45E-05 | 4.27E-03 |
| BCL2L15 | 1 | 20883 | 3.8678 | 5.49E-05 | 4.28E-03 |
| PGAP3 | 17 | 20883 | 3.8661 | 5.53E-05 | 4.29E-03 |
| HSPE1-MOB4 | 2 | 20883 | 3.8622 | 5.62E-05 | 4.34E-03 |
| IL2 | 4 | 20883 | 3.8614 | 5.64E-05 | 4.34E-03 |
| FNDC1 | 6 | 20883 | 3.8607 | 5.65E-05 | 4.34E-03 |
| MFN1 | 3 | 20883 | 3.8461 | 6.00E-05 | 4.58E-03 |
| PSORS1C2 | 6 | 20883 | 3.8433 | 6.07E-05 | 4.62E-03 |
| GPSM1 | 9 | 20883 | 3.8245 | 6.55E-05 | 4.96E-03 |
| QRICH1 | 3 | 20883 | 3.8206 | 6.66E-05 | 5.02E-03 |
| DCLRE1B | 1 | 20883 | 3.813 | 6.86E-05 | 5.16E-03 |
| GPR137 | 11 | 20883 | 3.8116 | 6.90E-05 | 5.17E-03 |
| SLC26A6 | 3 | 20883 | 3.8102 | 6.94E-05 | 5.18E-03 |
| CDC42SE2 | 5 | 20883 | 3.8028 | 7.15E-05 | 5.31E-03 |
| EFNA3 | 1 | 20883 | 3.7997 | 7.24E-05 | 5.36E-03 |
| ZBTB46 | 20 | 20883 | 3.7954 | 7.37E-05 | 5.43E-03 |
| FEN1 | 11 | 20883 | 3.7909 | 7.50E-05 | 5.51E-03 |
| ARHGEF2 | 1 | 20883 | 3.7885 | 7.58E-05 | 5.54E-03 |
| MAMSTR | 19 | 20883 | 3.7876 | 7.61E-05 | 5.54E-03 |
| SF3B1 | 2 | 20883 | 3.7859 | 7.66E-05 | 5.55E-03 |
| GALC | 14 | 20883 | 3.7854 | 7.67E-05 | 5.55E-03 |
| ACSL6 | 5 | 20883 | 3.7767 | 7.95E-05 | 5.73E-03 |
| ANKRD44 | 2 | 20883 | 3.7747 | 8.01E-05 | 5.75E-03 |
| AGER | 6 | 20883 | 3.7728 | 8.07E-05 | 5.77E-03 |
| C3orf84 | 3 | 20883 | 3.7648 | 8.34E-05 | 5.94E-03 |
| TEC | 4 | 20883 | 3.7527 | 8.75E-05 | 6.21E-03 |
| ZGLP1 | 19 | 20883 | 3.7495 | 8.86E-05 | 6.26E-03 |
| RNF5 | 6 | 20883 | 3.7408 | 9.17E-05 | 6.45E-03 |
| LSM14A | 19 | 20883 | 3.7403 | 9.19E-05 | 6.45E-03 |
| DUSP1 | 5 | 20883 | 3.7351 | 9.38E-05 | 6.56E-03 |
| DDAH2 | 6 | 20883 | 3.7309 | 9.54E-05 | 6.65E-03 |
| ZBTB38 | 3 | 20883 | 3.7195 | 9.98E-05 | 6.93E-03 |
| C11orf30 | 11 | 20883 | 3.7108 | 1.03E-04 | 7.15E-03 |
| SYNGR1 | 22 | 20883 | 3.7074 | 1.05E-04 | 7.21E-03 |
| SEMA3F | 3 | 20883 | 3.7018 | 1.07E-04 | 7.35E-03 |
| PAPD5 | 16 | 20883 | 3.6998 | 1.08E-04 | 7.38E-03 |
| VAMP3 | 1 | 20883 | 3.6822 | 1.16E-04 | 7.88E-03 |
| EFNA3 | 1 | 20883 | 3.6809 | 1.16E-04 | 7.89E-03 |
| CSNK2B | 6 | 20883 | 3.676 | 1.18E-04 | 8.02E-03 |
| SPNS1 | 16 | 20883 | 3.6745 | 1.19E-04 | 8.04E-03 |
| NDST2 | 10 | 20883 | 3.6552 | 1.28E-04 | 8.63E-03 |
| DGAT1 | 8 | 20883 | 3.6543 | 1.29E-04 | 8.64E-03 |
| UTS2 | 1 | 20883 | 3.6448 | 1.34E-04 | 8.93E-03 |
| DAP3 | 1 | 20883 | 3.639 | 1.37E-04 | 9.10E-03 |
| ZNF460 | 19 | 20883 | 3.6342 | 1.39E-04 | 9.24E-03 |
| LAMB2 | 3 | 20883 | 3.6286 | 1.43E-04 | 9.41E-03 |
| BOK | 2 | 20883 | 3.6251 | 1.44E-04 | 9.51E-03 |
| RP11-201K10.3 | 1 | 20883 | 3.6232 | 1.45E-04 | 9.54E-03 |
| UQCRC1 | 3 | 20883 | 3.6058 | 1.56E-04 | 1.02E-02 |
| GNB4 | 3 | 20883 | 3.6004 | 1.59E-04 | 1.03E-02 |
| CALCOCO2 | 17 | 20883 | 3.5898 | 1.65E-04 | 1.07E-02 |
| CD244 | 1 | 20883 | 3.5853 | 1.68E-04 | 1.09E-02 |
| LITAF | 16 | 20883 | 3.5748 | 1.75E-04 | 1.13E-02 |
| HLA-DQA2 | 6 | 20883 | 3.5747 | 1.75E-04 | 1.13E-02 |
| RMI2 | 16 | 20883 | 3.5642 | 1.82E-04 | 1.17E-02 |
| LBP | 20 | 20883 | 3.5606 | 1.85E-04 | 1.18E-02 |
| M6PR | 12 | 20883 | 3.557 | 1.88E-04 | 1.19E-02 |
| CRHR1 | 17 | 20883 | 3.5563 | 1.88E-04 | 1.19E-02 |
| LRRC25 | 19 | 20883 | 3.5425 | 1.98E-04 | 1.25E-02 |
| KRTCAP2 | 1 | 20883 | 3.5403 | 2.00E-04 | 1.26E-02 |
| BAD | 11 | 20883 | 3.5394 | 2.01E-04 | 1.26E-02 |
| UBAC2 | 13 | 20883 | 3.5377 | 2.02E-04 | 1.26E-02 |
| E2F3 | 6 | 20883 | 3.5345 | 2.04E-04 | 1.27E-02 |
| RP11-894J14.5 | 3 | 20883 | 3.5333 | 2.05E-04 | 1.28E-02 |
| TSPAN14 | 10 | 20883 | 3.5267 | 2.10E-04 | 1.30E-02 |
| EFNA1 | 1 | 20883 | 3.5242 | 2.12E-04 | 1.31E-02 |
| PRKAR2A | 3 | 20883 | 3.5178 | 2.18E-04 | 1.34E-02 |
| WIZ | 19 | 20883 | 3.5165 | 2.19E-04 | 1.34E-02 |
| ADAM15 | 1 | 20883 | 3.5122 | 2.22E-04 | 1.36E-02 |
| CAMK2G | 10 | 20883 | 3.508 | 2.26E-04 | 1.38E-02 |
| LACC1 | 13 | 20883 | 3.503 | 2.30E-04 | 1.40E-02 |
| PLAU | 10 | 20883 | 3.4951 | 2.37E-04 | 1.44E-02 |
| RASIP1 | 19 | 20883 | 3.4891 | 2.42E-04 | 1.46E-02 |
| RPL3 | 22 | 20883 | 3.4887 | 2.43E-04 | 1.46E-02 |
| TAB1 | 22 | 20883 | 3.4856 | 2.46E-04 | 1.47E-02 |
| NTN5 | 19 | 20883 | 3.4701 | 2.60E-04 | 1.56E-02 |
| LIMA1 | 12 | 20883 | 3.4663 | 2.64E-04 | 1.57E-02 |
| NOTCH2 | 1 | 20883 | 3.4644 | 2.66E-04 | 1.58E-02 |
| ADAM30 | 1 | 20883 | 3.4534 | 2.77E-04 | 1.64E-02 |
| ZSWIM8 | 10 | 20883 | 3.4511 | 2.79E-04 | 1.65E-02 |
| TMEM154 | 4 | 20883 | 3.4453 | 2.85E-04 | 1.68E-02 |
| CHSY3 | 5 | 20883 | 3.4433 | 2.87E-04 | 1.69E-02 |
| B3GALT6 | 1 | 20883 | 3.4421 | 2.89E-04 | 1.69E-02 |
| CTD-2369P2.12 | 19 | 20883 | 3.441 | 2.90E-04 | 1.69E-02 |
| CTC-432M15.3 | 5 | 20883 | 3.4402 | 2.91E-04 | 1.69E-02 |
| GHDC | 17 | 20883 | 3.4345 | 2.97E-04 | 1.72E-02 |
| CSF2RB | 22 | 20883 | 3.4304 | 3.01E-04 | 1.74E-02 |
| FNIP1 | 5 | 20883 | 3.4291 | 3.03E-04 | 1.74E-02 |
| HEATR3 | 16 | 20883 | 3.4136 | 3.21E-04 | 1.84E-02 |
| RBCK1 | 20 | 20883 | 3.4132 | 3.21E-04 | 1.84E-02 |
| CCNL2 | 1 | 20883 | 3.4117 | 3.23E-04 | 1.84E-02 |
| DLGAP4 | 20 | 20883 | 3.4101 | 3.25E-04 | 1.85E-02 |
| MYRF | 11 | 20883 | 3.4048 | 3.31E-04 | 1.88E-02 |
| MAPT | 17 | 20883 | 3.4011 | 3.36E-04 | 1.90E-02 |
| LTA | 6 | 20883 | 3.3915 | 3.48E-04 | 1.96E-02 |
| USMG5 | 10 | 20883 | 3.3862 | 3.54E-04 | 1.99E-02 |
| BOP1 | 8 | 20883 | 3.3862 | 3.54E-04 | 1.99E-02 |
| CCNY | 10 | 20883 | 3.3811 | 3.61E-04 | 2.02E-02 |
| LYZL2 | 10 | 20883 | 3.3805 | 3.62E-04 | 2.02E-02 |
| TRIP6 | 7 | 20883 | 3.3791 | 3.64E-04 | 2.02E-02 |
| DENND1B | 1 | 20883 | 3.3761 | 3.68E-04 | 2.04E-02 |
| ICOSLG | 21 | 20883 | 3.3749 | 3.69E-04 | 2.04E-02 |
| SPCS2 | 11 | 20883 | 3.3735 | 3.71E-04 | 2.05E-02 |
| HSPD1 | 2 | 20883 | 3.3721 | 3.73E-04 | 2.05E-02 |
| AC073569.1 | 12 | 20883 | 3.367 | 3.80E-04 | 2.08E-02 |
| CTC-454I21.3 | 19 | 20883 | 3.3508 | 4.03E-04 | 2.20E-02 |
| NAGLU | 17 | 20883 | 3.3481 | 4.07E-04 | 2.22E-02 |
| TNP2 | 16 | 20883 | 3.3451 | 4.11E-04 | 2.23E-02 |
| BTNL2 | 6 | 20883 | 3.3441 | 4.13E-04 | 2.24E-02 |
| RPL9 | 4 | 20883 | 3.3417 | 4.16E-04 | 2.25E-02 |
| ZNF639 | 3 | 20883 | 3.3372 | 4.23E-04 | 2.28E-02 |
| NEU1 | 6 | 20883 | 3.3348 | 4.27E-04 | 2.29E-02 |
| RAPGEF6 | 5 | 20883 | 3.3337 | 4.28E-04 | 2.30E-02 |
| FAM186A | 12 | 20883 | 3.3286 | 4.36E-04 | 2.32E-02 |
| RERG | 12 | 20883 | 3.3277 | 4.38E-04 | 2.32E-02 |
| CRB1 | 1 | 20883 | 3.3276 | 4.38E-04 | 2.32E-02 |
| STIP1 | 11 | 20883 | 3.3271 | 4.39E-04 | 2.32E-02 |
| C10orf55 | 10 | 20883 | 3.3262 | 4.40E-04 | 2.33E-02 |
| ABHD16A | 6 | 20883 | 3.3242 | 4.43E-04 | 2.34E-02 |
| RP11-178C3.1 | 17 | 20883 | 3.3206 | 4.49E-04 | 2.36E-02 |
| RP11-574K11.31 | 10 | 20883 | 3.3168 | 4.55E-04 | 2.39E-02 |
| SHE | 1 | 20883 | 3.3139 | 4.60E-04 | 2.40E-02 |
| ARIH2 | 3 | 20883 | 3.3089 | 4.68E-04 | 2.44E-02 |
| AKAP8L | 19 | 20883 | 3.3065 | 4.72E-04 | 2.45E-02 |
| C20orf166 | 20 | 20883 | 3.306 | 4.73E-04 | 2.45E-02 |
| KLB | 4 | 20883 | 3.3037 | 4.77E-04 | 2.47E-02 |
| IL10 | 1 | 20883 | 3.3007 | 4.82E-04 | 2.49E-02 |
| CAB39 | 2 | 20883 | 3.2967 | 4.89E-04 | 2.51E-02 |
| SETX | 9 | 20883 | 3.2955 | 4.91E-04 | 2.52E-02 |
| PEX13 | 2 | 20883 | 3.2907 | 5.00E-04 | 2.56E-02 |
| PRDX5 | 11 | 20883 | 3.2861 | 5.08E-04 | 2.59E-02 |
| LY6G6F | 6 | 20883 | 3.2844 | 5.11E-04 | 2.59E-02 |
| MEGT1 | 6 | 20883 | 3.2844 | 5.11E-04 | 2.59E-02 |
| GPRC5D | 12 | 20883 | 3.284 | 5.12E-04 | 2.59E-02 |
| DRD1 | 5 | 20883 | 3.2802 | 5.19E-04 | 2.62E-02 |
| RNFT1 | 17 | 20883 | 3.2794 | 5.20E-04 | 2.62E-02 |
| TCEB1 | 8 | 20883 | 3.2747 | 5.29E-04 | 2.65E-02 |
| PCGF6 | 10 | 20883 | 3.2726 | 5.33E-04 | 2.67E-02 |
| GON4L | 1 | 20883 | 3.2695 | 5.39E-04 | 2.69E-02 |
| NCOA1 | 2 | 20883 | 3.2597 | 5.58E-04 | 2.77E-02 |
| SMARCD1 | 12 | 20883 | 3.2596 | 5.58E-04 | 2.77E-02 |
| GDF15 | 19 | 20883 | 3.2569 | 5.63E-04 | 2.79E-02 |
| ZC3H7A | 16 | 20883 | 3.252 | 5.73E-04 | 2.83E-02 |
| ABHD17C | 15 | 20883 | 3.2472 | 5.83E-04 | 2.87E-02 |
| ZNF568 | 19 | 20883 | 3.2394 | 5.99E-04 | 2.94E-02 |
| SGK1 | 6 | 20883 | 3.2342 | 6.10E-04 | 2.99E-02 |
| MYCBP2 | 13 | 20883 | 3.2312 | 6.16E-04 | 3.01E-02 |
| ASGR2 | 17 | 20883 | 3.2278 | 6.24E-04 | 3.04E-02 |
| MED24 | 17 | 20883 | 3.2259 | 6.28E-04 | 3.04E-02 |
| DGKD | 2 | 20883 | 3.2259 | 6.28E-04 | 3.04E-02 |
| SYT11 | 1 | 20883 | 3.2253 | 6.29E-04 | 3.04E-02 |
| GPR65 | 14 | 20883 | 3.223 | 6.34E-04 | 3.06E-02 |
| BBS9 | 7 | 20883 | 3.2166 | 6.48E-04 | 3.12E-02 |
| KIF21B | 1 | 20883 | 3.2151 | 6.52E-04 | 3.13E-02 |
| ZNF585A | 19 | 20883 | 3.2143 | 6.54E-04 | 3.13E-02 |
| AC140061.12 | 12 | 20883 | 3.2107 | 6.62E-04 | 3.16E-02 |
| IL21 | 4 | 20883 | 3.2083 | 6.68E-04 | 3.18E-02 |
| ZNF420 | 19 | 20883 | 3.2079 | 6.69E-04 | 3.18E-02 |
| KIAA0907 | 1 | 20883 | 3.2034 | 6.79E-04 | 3.22E-02 |
| ATAD3C | 1 | 20883 | 3.2021 | 6.82E-04 | 3.23E-02 |
| UBTF | 17 | 20883 | 3.2006 | 6.86E-04 | 3.23E-02 |
| ZNF746 | 7 | 20883 | 3.1998 | 6.88E-04 | 3.23E-02 |
| KRTAP5-1 | 11 | 20883 | 3.1997 | 6.88E-04 | 3.23E-02 |
| ERRFI1 | 1 | 20883 | 3.1915 | 7.08E-04 | 3.32E-02 |
| CARD6 | 5 | 20883 | 3.1874 | 7.18E-04 | 3.35E-02 |
| WFDC8 | 20 | 20883 | 3.1817 | 7.32E-04 | 3.41E-02 |
| COL7A1 | 3 | 20883 | 3.1751 | 7.49E-04 | 3.48E-02 |
| ARIH2OS | 3 | 20883 | 3.1718 | 7.57E-04 | 3.51E-02 |
| PRKCQ | 10 | 20883 | 3.1704 | 7.61E-04 | 3.52E-02 |
| IL3 | 5 | 20883 | 3.1653 | 7.75E-04 | 3.58E-02 |
| XRRA1 | 11 | 20883 | 3.1577 | 7.95E-04 | 3.66E-02 |
| ITK | 5 | 20883 | 3.1397 | 8.46E-04 | 3.89E-02 |
| FERMT3 | 11 | 20883 | 3.1388 | 8.48E-04 | 3.89E-02 |
| ADCY3 | 2 | 20883 | 3.1233 | 8.94E-04 | 4.09E-02 |
| HAVCR1 | 5 | 20883 | 3.1191 | 9.07E-04 | 4.13E-02 |
| RPL29 | 3 | 20883 | 3.1187 | 9.08E-04 | 4.13E-02 |
| CCDC112 | 5 | 20883 | 3.1181 | 9.10E-04 | 4.13E-02 |
| CKB | 14 | 20883 | 3.1166 | 9.15E-04 | 4.14E-02 |
| AP001055.1 | 21 | 20883 | 3.1159 | 9.17E-04 | 4.14E-02 |
| SLC12A9 | 7 | 20883 | 3.1151 | 9.19E-04 | 4.14E-02 |
| METRNL | 17 | 20883 | 3.1138 | 9.23E-04 | 4.15E-02 |
| HGFAC | 4 | 20883 | 3.1096 | 9.37E-04 | 4.20E-02 |
| RAB19 | 7 | 20883 | 3.1065 | 9.47E-04 | 4.24E-02 |
| TNFRSF18 | 1 | 20883 | 3.1045 | 9.53E-04 | 4.26E-02 |
| C16orf47 | 16 | 20883 | 3.1032 | 9.57E-04 | 4.26E-02 |
| WNT3A | 1 | 20883 | 3.1031 | 9.57E-04 | 4.26E-02 |
| NFATC2IP | 16 | 20883 | 3.0963 | 9.80E-04 | 4.34E-02 |
| SLC35D1 | 1 | 20883 | 3.0928 | 9.91E-04 | 4.39E-02 |
| TRIM46 | 1 | 20883 | 3.0915 | 9.96E-04 | 4.39E-02 |
| IL2RA | 10 | 20883 | 3.0903 | 1.00E-03 | 4.40E-02 |
| PQLC1 | 18 | 20883 | 3.0897 | 1.00E-03 | 4.40E-02 |
| IRF6 | 1 | 20883 | 3.084 | 1.02E-03 | 4.47E-02 |
| OR8U1 | 11 | 20883 | 3.0833 | 1.02E-03 | 4.48E-02 |
| CDSN | 6 | 20883 | 3.0812 | 1.03E-03 | 4.50E-02 |
| FOSL2 | 2 | 20883 | 3.0783 | 1.04E-03 | 4.53E-02 |
| UBQLN4 | 1 | 20883 | 3.078 | 1.04E-03 | 4.53E-02 |
| KANSL1 | 17 | 20883 | 3.0766 | 1.05E-03 | 4.54E-02 |
| HLA-DQA1 | 6 | 20883 | 3.0731 | 1.06E-03 | 4.57E-02 |
| PLEKHH2 | 2 | 20883 | 3.0724 | 1.06E-03 | 4.57E-02 |
| DNAJC27 | 2 | 20883 | 3.0722 | 1.06E-03 | 4.57E-02 |
| NADK | 1 | 20883 | 3.0699 | 1.07E-03 | 4.58E-02 |
| PARK7 | 1 | 20883 | 3.0696 | 1.07E-03 | 4.58E-02 |
| KIF3A | 5 | 20883 | 3.0693 | 1.07E-03 | 4.58E-02 |
| NSF | 17 | 20883 | 3.0661 | 1.08E-03 | 4.62E-02 |
| NKX1-1 | 4 | 20883 | 3.0552 | 1.12E-03 | 4.78E-02 |
| ITLN1 | 1 | 20883 | 3.0546 | 1.13E-03 | 4.78E-02 |
| RNF145 | 5 | 20883 | 3.0524 | 1.14E-03 | 4.79E-02 |
| CX3CR1 | 3 | 20883 | 3.0522 | 1.14E-03 | 4.79E-02 |
| AGAP5 | 10 | 20883 | 3.0518 | 1.14E-03 | 4.79E-02 |
| WNT3 | 17 | 20883 | 3.0518 | 1.14E-03 | 4.79E-02 |
| SAPCD1 | 6 | 20883 | 3.0498 | 1.15E-03 | 4.79E-02 |
| C1orf74 | 1 | 20883 | 3.0494 | 1.15E-03 | 4.79E-02 |
| USP43 | 17 | 20883 | 3.0494 | 1.15E-03 | 4.79E-02 |
| AKAP8 | 19 | 20883 | 3.0484 | 1.15E-03 | 4.80E-02 |
| CCDC71 | 3 | 20883 | 3.0463 | 1.16E-03 | 4.82E-02 |
| RP11-293M10.1 | 14 | 20883 | 3.0442 | 1.17E-03 | 4.84E-02 |
| PLCG2 | 16 | 20883 | 3.0422 | 1.17E-03 | 4.87E-02 |
| ITGB6 | 2 | 20883 | 3.0385 | 1.19E-03 | 4.91E-02 |
| CPEB4 | 5 | 20883 | 3.0384 | 1.19E-03 | 4.91E-02 |
| ZNF423 | 16 | 20883 | 3.0363 | 1.20E-03 | 4.93E-02 |
| AAGAB | 15 | 20883 | 3.0354 | 1.20E-03 | 4.93E-02 |
| TDRD10 | 1 | 20883 | 3.0348 | 1.20E-03 | 4.93E-02 |
| IMPG2 | 3 | 20883 | 3.0319 | 1.22E-03 | 4.97E-02 |

**Table S3 The significant CD risk genes identified by FUSION**

| **GENE** | **CHR** | **TWAS.Z** | **TWAS.*P*** | ***P*_FDR_** |
| --- | --- | --- | --- | --- |
| SNX20 | 16 | 1.48E+01 | 1.62E-49 | 1.42E-45 |
| ATG16L1 | 2 | 1.21E+01 | 6.56E-34 | 2.89E-30 |
| NKD1 | 16 | -1.01E+01 | 8.65E-24 | 2.54E-20 |
| CARD9 | 9 | 9.11E+00 | 8.58E-20 | 1.89E-16 |
| SLC22A4 | 5 | -8.18E+00 | 2.74E-16 | 4.82E-13 |
| Y_RNA | 5 | -8.11E+00 | 5.18E-16 | 6.51E-13 |
| AC116366.6 | 5 | 8.12E+00 | 4.56E-16 | 6.51E-13 |
| DAG1 | 3 | 7.98E+00 | 1.51E-15 | 1.66E-12 |
| SLC22A5 | 5 | -7.90E+00 | 2.90E-15 | 2.83E-12 |
| RP11-327F22.1 | 16 | 7.53E+00 | 5.19E-14 | 4.57E-11 |
| HLA-S | 6 | 7.34E+00 | 2.21E-13 | 1.77E-10 |
| SEC16A | 9 | -7.22E+00 | 5.37E-13 | 3.94E-10 |
| TNFSF15 | 9 | -7.06E+00 | 1.61E-12 | 1.09E-09 |
| RP1-167A14.2 | 6 | -6.82E+00 | 9.10E-12 | 5.72E-09 |
| LNPEP | 5 | 6.57E+00 | 5.06E-11 | 2.97E-08 |
| RNASET2 | 6 | -6.54E+00 | 6.03E-11 | 3.31E-08 |
| LIME1 | 20 | -6.52E+00 | 7.22E-11 | 3.74E-08 |
| ERAP2 | 5 | 6.38E+00 | 1.74E-10 | 8.50E-08 |
| NPIPB7 | 16 | -6.34E+00 | 2.25E-10 | 1.04E-07 |
| NPIPB6 | 16 | 6.32E+00 | 2.67E-10 | 1.17E-07 |
| ADO | 10 | 6.25E+00 | 3.99E-10 | 1.67E-07 |
| ARFRP1 | 20 | -6.25E+00 | 4.21E-10 | 1.68E-07 |
| SULT1A2 | 16 | 6.16E+00 | 7.37E-10 | 2.70E-07 |
| AC007278.2 | 2 | -6.16E+00 | 7.17E-10 | 2.70E-07 |
| STMN3 | 20 | 6.13E+00 | 8.54E-10 | 3.00E-07 |
| ATP2A1 | 16 | 6.08E+00 | 1.17E-09 | 3.96E-07 |
| XXbac-BPG248L24.12 | 6 | 6.00E+00 | 1.99E-09 | 6.48E-07 |
| AAGAB | 15 | -5.96E+00 | 2.46E-09 | 7.70E-07 |
| ATXN2L | 16 | -5.94E+00 | 2.80E-09 | 7.70E-07 |
| SH2B1 | 16 | -5.95E+00 | 2.71E-09 | 7.70E-07 |
| TUFM | 16 | 5.95E+00 | 2.75E-09 | 7.70E-07 |
| ZBTB12 | 6 | 5.95E+00 | 2.68E-09 | 7.70E-07 |
| UBE2L3 | 22 | 5.84E+00 | 5.21E-09 | 1.39E-06 |
| RTEL1 | 20 | 5.78E+00 | 7.51E-09 | 1.94E-06 |
| CTD-2260A17.2 | 5 | -5.77E+00 | 7.97E-09 | 2.00E-06 |
| INPP5E | 9 | -5.74E+00 | 9.58E-09 | 2.34E-06 |
| UBA7 | 3 | 5.68E+00 | 1.39E-08 | 3.30E-06 |
| SDCCAG3 | 9 | -5.66E+00 | 1.52E-08 | 3.52E-06 |
| GSDMB | 17 | -5.65E+00 | 1.62E-08 | 3.65E-06 |
| IL18RAP | 2 | -5.62E+00 | 1.89E-08 | 4.16E-06 |
| RP4-591C20.9 | 20 | 5.52E+00 | 3.48E-08 | 7.47E-06 |
| BRD7 | 16 | -5.48E+00 | 4.20E-08 | 8.80E-06 |
| RPS6KA2 | 6 | -5.40E+00 | 6.58E-08 | 1.32E-05 |
| NDFIP1 | 5 | -5.41E+00 | 6.45E-08 | 1.32E-05 |
| EIF3C | 16 | 5.26E+00 | 1.44E-07 | 2.81E-05 |
| ORMDL3 | 17 | -5.23E+00 | 1.69E-07 | 3.23E-05 |
| IRGM | 5 | -5.17E+00 | 2.30E-07 | 4.30E-05 |
| RAVER1 | 19 | 5.13E+00 | 2.95E-07 | 5.41E-05 |
| ADCY7 | 16 | -5.12E+00 | 3.07E-07 | 5.51E-05 |
| IKZF3 | 17 | 5.09E+00 | 3.54E-07 | 6.23E-05 |
| RP4-583P15.16 | 20 | 5.08E+00 | 3.71E-07 | 6.40E-05 |
| SYT11 | 1 | -5.08E+00 | 3.87E-07 | 6.55E-05 |
| GALC | 14 | -5.02E+00 | 5.29E-07 | 8.78E-05 |
| IL12RB2 | 1 | -4.96E+00 | 6.97E-07 | 1.14E-04 |
| PLCL1 | 2 | 4.89E+00 | 1.01E-06 | 1.62E-04 |
| FAM183DP | 2 | 4.87E+00 | 1.09E-06 | 1.68E-04 |
| AC010883.5 | 2 | 4.87E+00 | 1.10E-06 | 1.68E-04 |
| RP11-1348G14.4 | 16 | -4.87E+00 | 1.11E-06 | 1.68E-04 |
| CLN3 | 16 | -4.74E+00 | 2.17E-06 | 3.24E-04 |
| FGFR1OP | 6 | 4.70E+00 | 2.60E-06 | 3.75E-04 |
| CTD-3184A7.4 | 20 | 4.70E+00 | 2.60E-06 | 3.75E-04 |
| NTN5 | 19 | 4.61E+00 | 3.99E-06 | 5.66E-04 |
| ADAM15 | 1 | -4.59E+00 | 4.44E-06 | 6.20E-04 |
| CISD1 | 10 | 4.58E+00 | 4.56E-06 | 6.24E-04 |
| IL27 | 16 | 4.58E+00 | 4.61E-06 | 6.24E-04 |
| APEH | 3 | 4.58E+00 | 4.70E-06 | 6.26E-04 |
| GBA | 1 | 4.54E+00 | 5.50E-06 | 7.11E-04 |
| MTX1P1 | 1 | 4.54E+00 | 5.50E-06 | 7.11E-04 |
| HLA-B | 6 | 4.53E+00 | 5.77E-06 | 7.36E-04 |
| SCAMP3 | 1 | 4.49E+00 | 7.13E-06 | 8.96E-04 |
| IPMK | 10 | 4.46E+00 | 8.28E-06 | 1.03E-03 |
| SP140 | 2 | -4.44E+00 | 8.85E-06 | 1.06E-03 |
| PBX2 | 6 | -4.45E+00 | 8.75E-06 | 1.06E-03 |
| AC034220.3 | 5 | 4.44E+00 | 8.92E-06 | 1.06E-03 |
| RBM6 | 3 | 4.42E+00 | 9.77E-06 | 1.15E-03 |
| PQLC1 | 18 | -4.41E+00 | 1.04E-05 | 1.20E-03 |
| THBS3 | 1 | 4.37E+00 | 1.26E-05 | 1.44E-03 |
| CCDC88B | 11 | 4.35E+00 | 1.37E-05 | 1.54E-03 |
| POLR2E | 19 | -4.33E+00 | 1.50E-05 | 1.67E-03 |
| MST1R | 3 | 4.28E+00 | 1.87E-05 | 2.05E-03 |
| SERBP1P3 | 3 | -4.28E+00 | 1.89E-05 | 2.05E-03 |
| CUL2 | 10 | -4.27E+00 | 1.97E-05 | 2.09E-03 |
| AP003774.1 | 11 | -4.27E+00 | 1.96E-05 | 2.09E-03 |
| TMEM258 | 11 | 4.26E+00 | 2.02E-05 | 2.12E-03 |
| SMIM19 | 8 | 4.24E+00 | 2.23E-05 | 2.31E-03 |
| GPR25 | 1 | -4.23E+00 | 2.35E-05 | 2.40E-03 |
| SNAPC4 | 9 | 4.22E+00 | 2.44E-05 | 2.47E-03 |
| SLC20A2 | 8 | 4.22E+00 | 2.47E-05 | 2.47E-03 |
| MST1 | 3 | 4.21E+00 | 2.55E-05 | 2.52E-03 |
| FKBPL | 6 | 4.20E+00 | 2.61E-05 | 2.55E-03 |
| ARHGAP15 | 2 | 4.19E+00 | 2.76E-05 | 2.67E-03 |
| RPS6KB1 | 17 | 4.19E+00 | 2.84E-05 | 2.72E-03 |
| RP11-263K19.6 | 1 | 4.18E+00 | 2.90E-05 | 2.74E-03 |
| CACNA1I | 22 | 4.16E+00 | 3.16E-05 | 2.96E-03 |
| ZMAT5 | 22 | 4.15E+00 | 3.34E-05 | 3.09E-03 |
| GBAP1 | 1 | -4.14E+00 | 3.50E-05 | 3.21E-03 |
| RFT1 | 3 | 4.13E+00 | 3.70E-05 | 3.36E-03 |
| GPSM1 | 9 | -4.10E+00 | 4.22E-05 | 3.79E-03 |
| CCDC116 | 22 | -4.08E+00 | 4.43E-05 | 3.88E-03 |
| PGAP3 | 17 | -4.09E+00 | 4.40E-05 | 3.88E-03 |
| ACSL6 | 5 | -4.08E+00 | 4.46E-05 | 3.88E-03 |
| CARD8 | 19 | 4.05E+00 | 5.12E-05 | 4.42E-03 |
| HEBP1 | 12 | 4.03E+00 | 5.63E-05 | 4.79E-03 |
| FAM234B | 12 | -4.03E+00 | 5.66E-05 | 4.79E-03 |
| RUSC1-AS1 | 1 | 4.01E+00 | 6.18E-05 | 5.18E-03 |
| PVALB | 22 | -4.00E+00 | 6.44E-05 | 5.34E-03 |
| DOCK7 | 1 | -3.98E+00 | 6.90E-05 | 5.64E-03 |
| RNF5 | 6 | -3.98E+00 | 6.92E-05 | 5.64E-03 |
| CD244 | 1 | -3.97E+00 | 7.28E-05 | 5.87E-03 |
| ICAM3 | 19 | 3.94E+00 | 8.12E-05 | 6.43E-03 |
| AC114271.2 | 19 | 3.94E+00 | 8.12E-05 | 6.43E-03 |
| TAC3 | 12 | 3.93E+00 | 8.54E-05 | 6.66E-03 |
| SLC26A6 | 3 | 3.93E+00 | 8.55E-05 | 6.66E-03 |
| FDPS | 1 | -3.90E+00 | 9.66E-05 | 7.45E-03 |
| ABHD16A | 6 | 3.89E+00 | 9.88E-05 | 7.49E-03 |
| CTC-429L19.3 | 19 | -3.89E+00 | 9.88E-05 | 7.49E-03 |
| SPNS1 | 16 | 3.88E+00 | 1.05E-04 | 7.89E-03 |
| PPIF | 10 | -3.84E+00 | 1.22E-04 | 9.09E-03 |
| ATG4C | 1 | -3.83E+00 | 1.29E-04 | 9.54E-03 |
| DENND1B | 1 | 3.82E+00 | 1.33E-04 | 9.75E-03 |
| IFITM2 | 11 | 3.82E+00 | 1.36E-04 | 9.89E-03 |
| XXcos-LUCA16.1 | 3 | 3.80E+00 | 1.44E-04 | 1.04E-02 |
| NONOP2 | 2 | -3.79E+00 | 1.49E-04 | 1.07E-02 |
| ZBTB38 | 3 | 3.76E+00 | 1.68E-04 | 1.19E-02 |
| CASC3 | 17 | -3.73E+00 | 1.89E-04 | 1.33E-02 |
| NAAA | 4 | 3.71E+00 | 2.05E-04 | 1.43E-02 |
| BMS1P4 | 10 | 3.70E+00 | 2.16E-04 | 1.50E-02 |
| NEU1 | 6 | 3.66E+00 | 2.56E-04 | 1.76E-02 |
| C4B | 6 | -3.64E+00 | 2.68E-04 | 1.83E-02 |
| LIMA1 | 12 | 3.64E+00 | 2.74E-04 | 1.83E-02 |
| IER3 | 6 | -3.64E+00 | 2.71E-04 | 1.83E-02 |
| HKR1 | 19 | 3.64E+00 | 2.75E-04 | 1.83E-02 |
| FADS1 | 11 | 3.63E+00 | 2.79E-04 | 1.84E-02 |
| SGF29 | 16 | -3.63E+00 | 2.80E-04 | 1.84E-02 |
| AC093690.1 | 2 | 3.63E+00 | 2.83E-04 | 1.84E-02 |
| HLA-U | 6 | -3.63E+00 | 2.86E-04 | 1.85E-02 |
| DND1P1 | 17 | -3.62E+00 | 2.99E-04 | 1.92E-02 |
| RP11-574K11.24 | 10 | 3.61E+00 | 3.03E-04 | 1.93E-02 |
| GATS | 7 | -3.61E+00 | 3.11E-04 | 1.97E-02 |
| RP11-263K19.4 | 1 | 3.60E+00 | 3.21E-04 | 2.02E-02 |
| RPL9 | 4 | 3.59E+00 | 3.26E-04 | 2.02E-02 |
| MAPK8IP1P2 | 17 | -3.59E+00 | 3.26E-04 | 2.02E-02 |
| TXNL4A | 18 | -3.59E+00 | 3.30E-04 | 2.03E-02 |
| C2 | 6 | -3.59E+00 | 3.32E-04 | 2.03E-02 |
| RP11-707O23.1 | 17 | -3.58E+00 | 3.37E-04 | 2.04E-02 |
| LINC02210 | 17 | -3.58E+00 | 3.46E-04 | 2.08E-02 |
| SKIV2L | 6 | 3.57E+00 | 3.53E-04 | 2.11E-02 |
| EGFL8 | 6 | -3.57E+00 | 3.57E-04 | 2.12E-02 |
| IRF6 | 1 | -3.56E+00 | 3.74E-04 | 2.16E-02 |
| PGPEP1 | 19 | -3.56E+00 | 3.69E-04 | 2.16E-02 |
| MFN1 | 3 | -3.55E+00 | 3.79E-04 | 2.16E-02 |
| KANSL1-AS1 | 17 | -3.56E+00 | 3.74E-04 | 2.16E-02 |
| PLEKHM1 | 17 | 3.56E+00 | 3.75E-04 | 2.16E-02 |
| HLA-DOB | 6 | -3.56E+00 | 3.77E-04 | 2.16E-02 |
| RP11-798G7.6 | 17 | 3.55E+00 | 3.80E-04 | 2.16E-02 |
| MAPK8IP1P1 | 17 | -3.55E+00 | 3.90E-04 | 2.20E-02 |
| RP11-259G18.3 | 17 | -3.54E+00 | 3.93E-04 | 2.20E-02 |
| HEATR3 | 16 | -3.54E+00 | 4.00E-04 | 2.23E-02 |
| HMP19 | 5 | -3.53E+00 | 4.17E-04 | 2.31E-02 |
| UBE2Q2P2 | 15 | 3.52E+00 | 4.33E-04 | 2.38E-02 |
| MUC1 | 1 | 3.50E+00 | 4.57E-04 | 2.50E-02 |
| CECR6 | 22 | -3.50E+00 | 4.67E-04 | 2.54E-02 |
| MEIKIN | 5 | -3.50E+00 | 4.72E-04 | 2.55E-02 |
| DTX4 | 11 | 3.49E+00 | 4.79E-04 | 2.57E-02 |
| GMEB2 | 20 | -3.46E+00 | 5.41E-04 | 2.88E-02 |
| PARK7 | 1 | -3.45E+00 | 5.58E-04 | 2.96E-02 |
| ITLN1 | 1 | 3.44E+00 | 5.82E-04 | 3.05E-02 |
| C5orf56 | 5 | 3.44E+00 | 5.80E-04 | 3.05E-02 |
| AC007193.9 | 19 | 3.43E+00 | 6.01E-04 | 3.13E-02 |
| PPP5C | 19 | 3.43E+00 | 6.12E-04 | 3.17E-02 |
| STARD3 | 17 | -3.42E+00 | 6.17E-04 | 3.17E-02 |
| C4A | 6 | 3.42E+00 | 6.25E-04 | 3.20E-02 |
| UQCR10 | 22 | 3.42E+00 | 6.34E-04 | 3.22E-02 |
| RBCK1 | 20 | -3.41E+00 | 6.38E-04 | 3.23E-02 |
| HLA-DPA1 | 6 | -3.41E+00 | 6.46E-04 | 3.25E-02 |
| LRRC37A4P | 17 | 3.41E+00 | 6.57E-04 | 3.28E-02 |
| TAP2 | 6 | 3.40E+00 | 6.65E-04 | 3.30E-02 |
| FADS2 | 11 | 3.40E+00 | 6.85E-04 | 3.37E-02 |
| ANXA6 | 5 | 3.40E+00 | 6.85E-04 | 3.37E-02 |
| FNIP1 | 5 | -3.38E+00 | 7.22E-04 | 3.53E-02 |
| AF064858.8 | 21 | 3.37E+00 | 7.55E-04 | 3.67E-02 |
| WDR6 | 3 | 3.35E+00 | 8.10E-04 | 3.91E-02 |
| EDN3 | 20 | -3.35E+00 | 8.19E-04 | 3.92E-02 |
| FLOT1 | 6 | -3.35E+00 | 8.18E-04 | 3.92E-02 |
| SDF4 | 1 | -3.34E+00 | 8.24E-04 | 3.92E-02 |
| RNFT1 | 17 | -3.34E+00 | 8.35E-04 | 3.95E-02 |
| CREM | 10 | -3.34E+00 | 8.41E-04 | 3.96E-02 |
| MON1A | 3 | 3.33E+00 | 8.73E-04 | 4.08E-02 |
| CCDC102A | 16 | -3.32E+00 | 9.00E-04 | 4.19E-02 |
| RP11-973H7.1 | 18 | 3.32E+00 | 9.13E-04 | 4.23E-02 |
| TMEM229B | 14 | -3.31E+00 | 9.29E-04 | 4.28E-02 |
| CCDC122 | 13 | -3.31E+00 | 9.42E-04 | 4.32E-02 |
| FAM117B | 2 | -3.30E+00 | 9.57E-04 | 4.36E-02 |
| PFKFB4 | 3 | 3.29E+00 | 9.96E-04 | 4.52E-02 |
| ANKRD55 | 5 | 3.29E+00 | 1.01E-03 | 4.55E-02 |
| RP11-876N24.1 | 16 | -3.29E+00 | 1.02E-03 | 4.55E-02 |
| RP4-605O3.4 | 12 | -3.29E+00 | 1.02E-03 | 4.55E-02 |
| NELFE | 6 | -3.27E+00 | 1.06E-03 | 4.66E-02 |
| ATF6B | 6 | -3.27E+00 | 1.06E-03 | 4.66E-02 |
| RP11-219E7.1 | 14 | -3.27E+00 | 1.06E-03 | 4.66E-02 |
| AC020571.3 | 2 | -3.27E+00 | 1.09E-03 | 4.77E-02 |
| AGAP12P | 10 | -3.26E+00 | 1.11E-03 | 4.83E-02 |
| TM4SF19 | 3 | -3.25E+00 | 1.14E-03 | 4.94E-02 |

**Table S4 The intersection genes between cross-tissue and single-tissue analyses**

| **GENE** | **CHR** | **TWAS.Z** | **TWAS.*P*** | ***P*_FDR_** |
| --- | --- | --- | --- | --- |
| ATG16L1 | 2 | 12.14 | 6.56E-34 | 2.89E-30 |
| PLCL1 | 2 | 4.89 | 1.01E-06 | 1.62E-04 |
| RPL9 | 4 | 3.59 | 3.26E-04 | 2.02E-02 |
| RP11-973H7.1 | 18 | 3.32 | 9.13E-04 | 4.23E-02 |

**Table S5 The results of FOCUS precision positioning**

| **GENE** | **CHR** | **Ldregion pop1** | **Twas z pop1** | **Pips pop1** | **In cred set pop1** |
| --- | --- | --- | --- | --- | --- |
| NKD1 | 16 | 16:49008253-16:52035823 | -9.86 | 1 | 1 |
| Y_RNA | 5 | 5:129519025-5:132139647 | -8.16 | 0.996 | 1 |
| TNFSF15 | 9 | 9:117019801-9:117921871 | -6.73 | 1 | 1 |
| HLA-DRB1 | 6 | 6:32682664-6:33236497 | -5.99 | 0.999 | 1 |
| STK19 | 6 | 6:30798168-6:31570931 | -5.86 | 0.91 | 1 |
| AAGAB | 15 | 15:67094767-15:69017999 | -5.71 | 1 | 1 |
| NDFIP1 | 5 | 5:140645971-5:142980181 | -5.25 | 0.999 | 1 |
| ADCY7 | 16 | 16:49008253-16:52035823 | -5.06 | 0.826 | 1 |
| IRGM | 5 | 5:148662633-5:150561298 | -5.03 | 1 | 1 |
| IL12RB2 | 1 | 1:66939404-1:68476806 | -4.74 | 0.997 | 1 |
| SP140 | 2 | 2:229370787-2:231842809 | -4.28 | 0.958 | 1 |
| NONOP2 | 2 | 2:60292000-2:62428117 | -4.26 | 0.955 | 1 |
| POLR2E | 19 | 19:610729-19:2098237 | -4.23 | 0.904 | 1 |
| CUL2 | 10 | 10:35109355-10:36572508 | -4.14 | 0.914 | 1 |
| PPIF | 10 | 10:80877086-10:82414679 | -3.5 | 0.929 | 1 |
| MPC1 | 6 | 6:167024733-6:168042835 | -3.15 | 0.834 | 1 |
| CACNA1I | 22 | 22:39307894-22:40545595 | 4.04 | 0.936 | 1 |
| RAVER1 | 19 | 19:9238548-19:11284028 | 4.46 | 0.961 | 1 |
| ENSG00000234936.1 | 2 | 2:43309590-2:44313803 | 4.76 | 0.998 | 1 |
| SPATA6L | 9 | 9:4885082-9:6557589 | 4.83 | 0.995 | 1 |
| NAGLU | 17 | 17:39900765-17:41772005 | 4.93 | 0.994 | 1 |
| ENSG00000271581.1 | 6 | 6:30798168-6:31570931 | 5.52 | 0.948 | 1 |
| UBE2L3 | 22 | 22:19912358-22:22357325 | 5.74 | 0.999 | 1 |
| ADO | 10 | 10:63341695-10:65793994 | 6.1 | 1 | 1 |
| HLA-S | 6 | 6:31571337-6:32682664 | 6.48 | 0.835 | 1 |
| LNPEP | 5 | 5:95963902-5:97314960 | 6.72 | 0.869 | 1 |
| DAG1 | 3 | 3:49317338-3:51830565 | 7.97 | 1 | 1 |
| CARD9 | 9 | 9:137589364-9:141124247 | 8.96 | 1 | 1 |
| ATG16L1 | 2 | 2:233550145-2:235150987 | 12.5 | 1 | 1 |
| SNX20 | 16 | 16:49008253-16:52035823 | 14.3 | 1 | 1 |


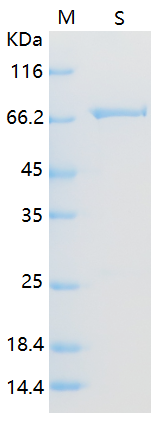

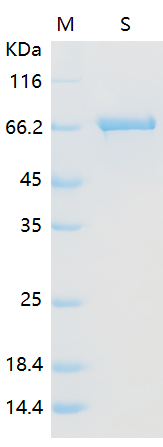
**Figure S1** **ATG16L1 wild type Figure S2** **ATG16L1 T300A mutant**

**SDS-PAGE Result**

Lane M：Protein Marker; Lane S：Final Sample

**Figure S3 Venn diagram of the intersection of four genetic analysis methods**


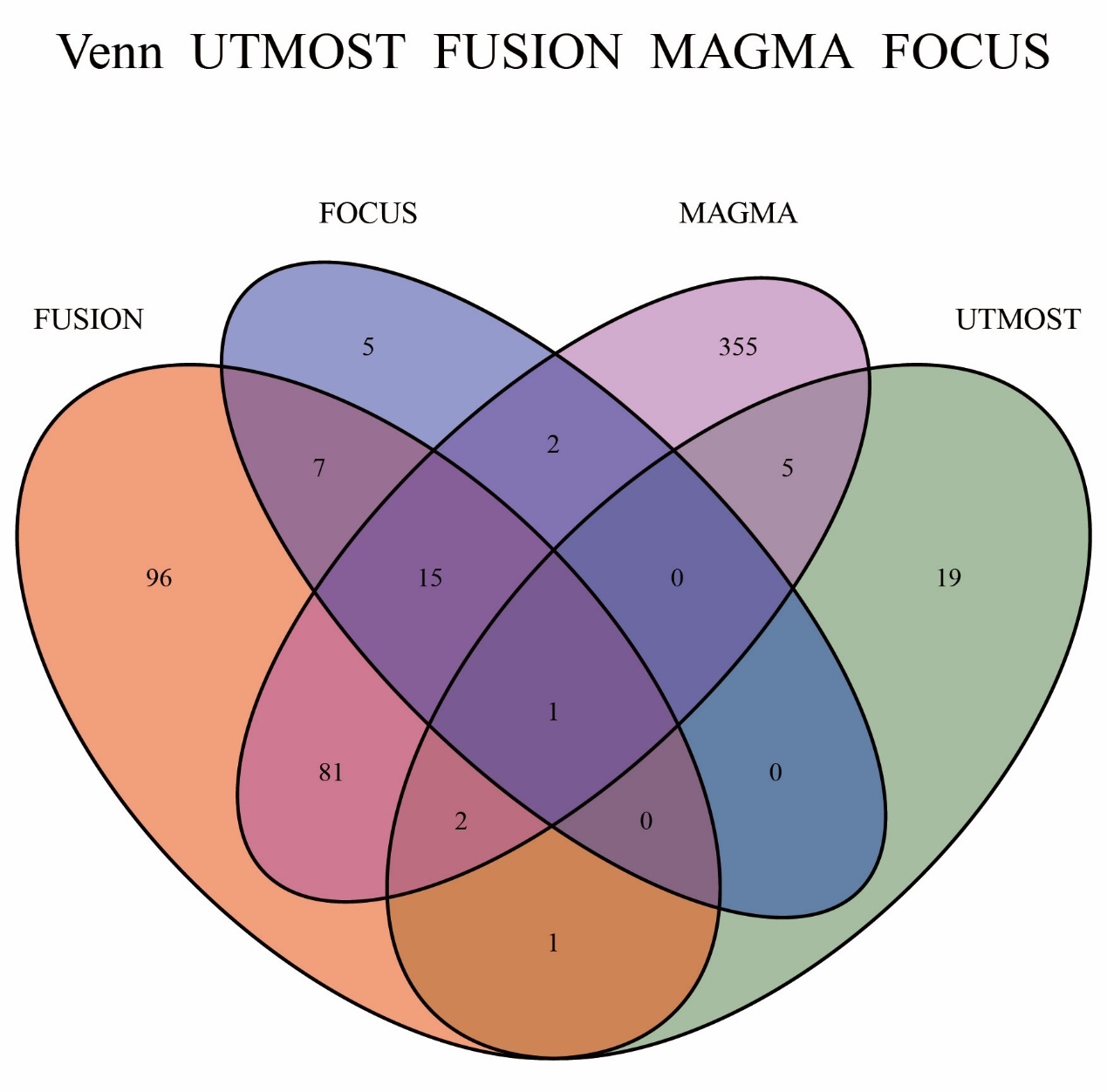

Supplement: Supplementary file 1 [file Table_1.docx]
