# Supplementary figures and images for: The role of ATG16L1 in Crohn’s disease and the structural alteration mechanisms and functional consequences of the rs2241880 variant
Source: Front Med (Lausanne). 2025 Oct 3;12:1656575. doi: 10.3389/fmed.2025.1656575 (PMC12531253; doi:10.3389/fmed.2025.1656575)

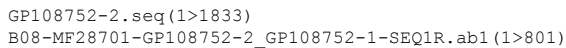

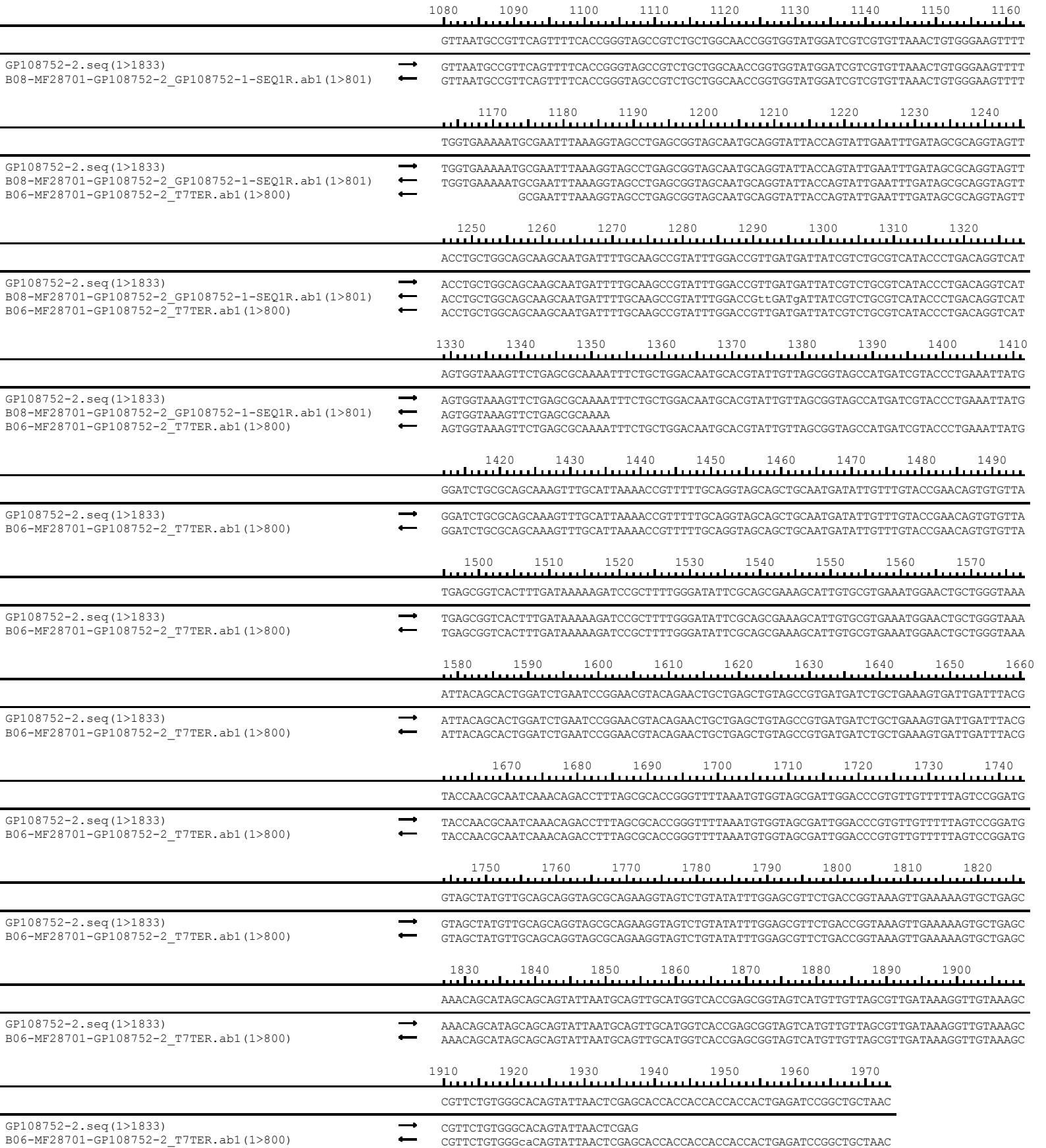

Supplement: Supplementary file 3 [file Data_Sheet_2.pdf]
