# Supplementary material for: The role of ATG16L1 in Crohn’s disease and the structural alteration mechanisms and functional consequences of the rs2241880 variant
Source: Front Med (Lausanne). 2025 Oct 3;12:1656575. doi: 10.3389/fmed.2025.1656575 (PMC12531253; doi:10.3389/fmed.2025.1656575)
Supplement: Supplementary file 2 [file Data_Sheet_1.pdf]

```
                                                             10        20        30        40        50        60        70        80
                                                   CTAGAAATAATTTTGTTTAACTTTAAGAAGGAGATATACCATGGGCAGCAGCCATCATCATCATCATCACAGCAGCGGCCTGGT
G05-F26342-GP108752-1_PET-SEQR.ab1(1>799)       →  CTAGAAATAATTTTGTTTAACTTTAAGAAGGAGATATACCATGGGCAGCAGCCATCATCATCATCATCACAGCAGCGGCCTGGT

                                                       90        100       110       120       130       140       150       160
                                                   GCCGCGCGGCAGCCATATGAGCAGCGGTCTGCGTGCAGCAGATTTTCCGCGTTGGAAACGTCATATTTCAGAACAGCTGCGTCG
G05-F26342-GP108752-1_PET-SEQR.ab1(1>799)       →  GCCGCGCGGCAGCCATATGAGCAGCGGTCTGCGTGCAGCAGATTTTCCGCGTTGGAAACGTCATATTTCAGAACAGCTGCGTCG
GP108752-1.seq(1>1833)                          →               CATATGAGCAGCGGTCTGCGTGCAGCAGATTTTCCGCGTTGGAAACGTCATATTTCAGAACAGCTGCGTCG

                                                   170       180       190       200       210       220       230       240       250
                                                   TCGTGATCGTCTGCAGCGTCAGGCATTTGAAGAAATTATTCTGCAGTACAACAAACTGCTGGAAAAGAGCGATCTGCATAGCGT
G05-F26342-GP108752-1_PET-SEQR.ab1(1>799)       →  TCGTGATCGTCTGCAGCGTCAGGCATTTGAAGAAATTATTCTGCAGTACAACAAACTGCTGGAAAAGAGCGATCTGCATAGCGT
GP108752-1.seq(1>1833)                          →  TCGTGATCGTCTGCAGCGTCAGGCATTTGAAGAAATTATTCTGCAGTACAACAAACTGCTGGAAAAGAGCGATCTGCATAGCGT

                                                         260       270       280       290       300       310       320       330
                                                   TCTGGCACAGAAACTGCAGGCAGAAAAACATGATGTTCCGAATCGTCATGAAATTAGTCCGGGTCATGATGGCACCTGGAATGA
G05-F26342-GP108752-1_PET-SEQR.ab1(1>799)       →  TCTGGCACAGAAACTGCAGGCAGAAAAACATGATGTTCCGAATCGTCATGAAATTAGTCCGGGTCATGATGGCACCTGGAATGA
GP108752-1.seq(1>1833)                          →  TCTGGCACAGAAACTGCAGGCAGAAAAACATGATGTTCCGAATCGTCATGAAATTAGTCCGGGTCATGATGGCACCTGGAATGA

                                                     340       350       360       370       380       390       400       410       420
                                                   TAATCAGCTGCAAGAAATGGCCCAGCTGCGTATTAAACATCAAGAAGAACTGACCGAACTGCATAAAAAGCGTGGTGAACTGGC
G05-F26342-GP108752-1_PET-SEQR.ab1(1>799)       →  TAATCAGCTGCAAGAAATGGCCCAGCTGCGTATTAAACATCAAGAAGAACTGACCGAACTGCATAAAAAGCGTGGTGAACTGGC
GP108752-1.seq(1>1833)                          →  TAATCAGCTGCAAGAAATGGCCCAGCTGCGTATTAAACATCAAGAAGAACTGACCGAACTGCATAAAAAGCGTGGTGAACTGGC

                                                             430       440       450       460       470       480       490       500
                                                   ACAGCTGGTTATTGATCTGAATAATCAGATGCAGCGTAAAGATCGTGAAATGCAGATGAACGAAGCAAAAATTGCAGAATGCCT
G05-F26342-GP108752-1_PET-SEQR.ab1(1>799)       →  ACAGCTGGTTATTGATCTGAATAATCAGATGCAGCGTAAAGATCGTGAAATGCAGATGAACGAAGCAAAAATTGCAGAATGCCT
GP108752-1.seq(1>1833)                          →  ACAGCTGGTTATTGATCTGAATAATCAGATGCAGCGTAAAGATCGTGAAATGCAGATGAACGAAGCAAAAATTGCAGAATGCCT

                                                       510       520       530       540       550       560       570       580
                                                   GCAGACCATTAGCGATCTGGAAACCGAATGTCTGGATCTGCGTACCAAACTGTGTGATCTGGAACGTGCAAATCAGACCCTGAA
G05-F26342-GP108752-1_PET-SEQR.ab1(1>799)       →  GCAGACCATTAGCGATCTGGAAACCGAATGTCTGGATCTGCGTACCAAACTGTGTGATCTGGAACGTGCAAATCAGACCCTGAA
GP108752-1.seq(1>1833)                          →  GCAGACCATTAGCGATCTGGAAACCGAATGTCTGGATCTGCGTACCAAACTGTGTGATCTGGAACGTGCAAATCAGACCCTGAA
G06-F26342-GP108752-1_GP108752-1-SEQ1R.ab1(1>799) ← TCTGCGTACCAAACTGTGTGATCTGGAACGTGCAAATCAGACCCTGAA

                                                   590       600       610       620       630       640       650       660       670
                                                   AGATGAATATGATGCACTGCAGATTACCTTTACCGCACTGGAAGGTAAACTGCGTAAAACCACCGAAGAAAATCAAGAACTGGT
G05-F26342-GP108752-1_PET-SEQR.ab1(1>799)       →  AGATGAATATGATGCACTGCAGATTACCTTTACCGCACTGGAAGGTAAACTGCGTAAAACCACCGAAGAAAATCAAGAACTGGT
GP108752-1.seq(1>1833)                          →  AGATGAATATGATGCACTGCAGATTACCTTTACCGCACTGGAAGGTAAACTGCGTAAAACCACCGAAGAAAATCAAGAACTGGT
G06-F26342-GP108752-1_GP108752-1-SEQ1R.ab1(1>799) ← AGATGAATATGATGCACTGCAGATTACCTTTACCGCACTGGAAGGTAAACTGCGTAAAACCACCGAAGAAAATCAAGAACTGGT

                                                         680       690       700       710       720       730       740       750
                                                   TACCCGTTGGATGGCCGAAAAAGCACAAGAGGCAAATCGTCTGAATGCCGAAAATGAAAAAGATAGCCGTCGTCGTCAGGCACG
G05-F26342-GP108752-1_PET-SEQR.ab1(1>799)       →  TACCCGTTGGATGGCCGAAAAAGCACAAGAGGCAAATCGTCTGAATGCCGAAAATGAAAAAGATAGCCGTCGTCGTCAGGCACG
GP108752-1.seq(1>1833)                          →  TACCCGTTGGATGGCCGAAAAAGCACAAGAGGCAAATCGTCTGAATGCCGAAAATGAAAAAGATAGCCGTCGTCGTCAGGCACG
G06-F26342-GP108752-1_GP108752-1-SEQ1R.ab1(1>799) ← TACCCGTTGGATGGCCGAAAAAGCACAAGAGGCAAATCGTCTGAATGCCGAAAATGAAAAAGATAGCCGTCGTCGTCAGGCACG

                                                     760       770       780       790       800       810       820       830       840
                                                   TCTGCAGAAAGAACTGGCAGAAGCAGCAAAAGAACCGCTGCCGGTTGAACAGGATGATGATATTGAAGTTATTGTGGATGAAAC
G05-F26342-GP108752-1_PET-SEQR.ab1(1>799)       →  TCTGCAGAAAGAACTGGCAGAAGCAGCAAAAGAACCGCTGCCG
GP108752-1.seq(1>1833)                          →  TCTGCAGAAAGAACTGGCAGAAGCAGCAAAAGAACCGCTGCCGGTTGAACAGGATGATGATATTGAAGTTATTGTGGATGAAAC
G06-F26342-GP108752-1_GP108752-1-SEQ1R.ab1(1>799) ← TCTGCAGAAAGAACTGGCAGAAGCAGCAAAAGAACCGCTGCCGGTTGAACAGGATGATGATATTGAAGTTATTGTGGATGAAAC

                                                             850       860       870       880       890       900       910       920
                                                   CAGCGATCATACAGAAGAAACCAGTCCGGTTCGTGCAATTAGCCGTGCAGCAACCAAACGTCTGAGCCAGCCTGCCGGTGGTCT
GP108752-1.seq(1>1833)                          →  CAGCGATCATACAGAAGAAACCAGTCCGGTTCGTGCAATTAGCCGTGCAGCAACCAAACGTCTGAGCCAGCCTGCCGGTGGTCT
G06-F26342-GP108752-1_GP108752-1-SEQ1R.ab1(1>799) ← CAGCGATCATACAGAAGAAACCAGTCCGGTTCGTGCAATTAGCCGTGCAGCAACCAAACGTCTGAGCCAGCCTGCCGGTGGTCT

                                                       930       940       950       960       970       980       990       1000
                                                   GCTGGATAGCATTACCAACATTTTTGGTCGTCGTAGCGTTAGCAGCTTTCCGGTTCCGCAGGATAATGTTGATACCCATCCTGG
GP108752-1.seq(1>1833)                          →  GCTGGATAGCATTACCAACATTTTTGGTCGTCGTAGCGTTAGCAGCTTTCCGGTTCCGCAGGATAATGTTGATACCCATCCTGG
G06-F26342-GP108752-1_GP108752-1-SEQ1R.ab1(1>799) ← GCTGGATAGCATTACCAACATTTTTGGTCGTCGTAGCGTTAGCAGCTTTCCGGTTCCGCAGGATAATGTTGATACCCATCCTGG

                                                   1010      1020      1030      1040      1050      1060      1070      1080      1090
                                                   TAGCGGTAAAGAAGTTCGCGTTCCGGCAACCGCACTGTGTGTTTTTGATGCACATGATGGTGAAGTTAATGCCGTTCAGTTTTC
GP108752-1.seq(1>1833)                          →  TAGCGGTAAAGAAGTTCGCGTTCCGGCAACCGCACTGTGTGTTTTTGATGCACATGATGGTGAAGTTAATGCCGTTCAGTTTTC
G06-F26342-GP108752-1_GP108752-1-SEQ1R.ab1(1>799) ← TAGCGGTAAAGAAGTTCGCGTTCCGGCAACCGCACTGTGTGTTTTTGATGCACATGATGGTGAAGTTAATGCCGTTCAGTTTTC
```

```
                                                      1100      1110      1120      1130      1140      1150      1160      1170
                                                      ACCGGGTAGCCGTCTGCTGGCAACCGGTGGTATGGATCGTCGTGTTAAACTGTGGGAAGTTTTTGGTGAAAAATGCGAATTTAA
GP108752-1.seq(1>1833)                             →  ACCGGGTAGCCGTCTGCTGGCAACCGGTGGTATGGATCGTCGTGTTAAACTGTGGGAAGTTTTTGGTGAAAAATGCGAATTTAA
G06-F26342-GP108752-1_GP108752-1-SEQ1R.ab1(1>799)  ←  ACCGGGTAGCCGTCTGCTGGCAACCGGTGGTATGGATCGTCGTGTTAAACTGTGGGAAGTTTTTGGTGAAAAATGCGAATTTAA
G04-F26342-GP108752-1_T7TER.ab1(1>799)             ←                                                                        AAAAATGCGAATTTAA

                                                      1180      1190      1200      1210      1220      1230      1240      1250      1260
                                                      AGGTAGCCTGAGCGGTAGCAATGCAGGTATTACCAGTATTGAATTTGATAGCGCAGGTAGTTACCTGCTGGCAGCAAGCAATGA
GP108752-1.seq(1>1833)                             →  AGGTAGCCTGAGCGGTAGCAATGCAGGTATTACCAGTATTGAATTTGATAGCGCAGGTAGTTACCTGCTGGCAGCAAGCAATGA
G06-F26342-GP108752-1_GP108752-1-SEQ1R.ab1(1>799)  ←  AGGTAGCCTGAGCGGTAGCAATGCAGGTATTACCAGTATTGAATTTGATAGCGCAGGTAGTTACCTGCTGGCAGCAAGCAATGA
G04-F26342-GP108752-1_T7TER.ab1(1>799)             ←  AGGTAGCcTGAGCGGTAGCAATGCAGGTATTACCAGTATTGAATTTGATAGCGCAGGTAGTTACCTGCTGGCAGCAAGCAATGA

                                                      1270      1280      1290      1300      1310      1320      1330      1340
                                                      TTTTGCAAGCCGTATTTGGACCGTTGATGATTATCGTCTGCGTCATACCCTGACAGGTCATAGTGGTAAAGTTCTGAGCGCAAA
GP108752-1.seq(1>1833)                             →  TTTTGCAAGCCGTATTTGGACCGTTGATGATTATCGTCTGCGTCATACCCTGACAGGTCATAGTGGTAAAGTTCTGAGCGCAAA
G06-F26342-GP108752-1_GP108752-1-SEQ1R.ab1(1>799)  ←  TTTTGCAAGCCGTATTTGGACCGTTGATGATTATCGTCTGCGTCATACCCTGACAGGTCATAGTGGTAAAGTTCTGAGC
G04-F26342-GP108752-1_T7TER.ab1(1>799)             ←  TTTTGCAAGCCGTATTTGGACCGTTGATGATTATCGTCTGCGTCATACCCTGACAGGTCATAGTGGTAAAGTTCTGAGCGCAAA

                                                      1350      1360      1370      1380      1390      1400      1410      1420
                                                      ATTTCTGCTGGACAATGCACGTATTGTTAGCGGTAGCCATGATCGTACCCTGAAATTATGGGATCTGCGCAGCAAAGTTTGCAT
GP108752-1.seq(1>1833)                             →  ATTTCTGCTGGACAATGCACGTATTGTTAGCGGTAGCCATGATCGTACCCTGAAATTATGGGATCTGCGCAGCAAAGTTTGCAT
G04-F26342-GP108752-1_T7TER.ab1(1>799)             ←  ATTTCTGCTGGACAATGCACGTATTGTTAGCGGTAGCCATGATCGTACCCTGAAATTATGGGATCTGCGCAGCAAAGTTTGCAT

                                                      1430      1440      1450      1460      1470      1480      1490      1500      1510
                                                      TAAAACCGTTTTTGCAGGTAGCAGCTGCAATGATATTGTTTGTACCGAACAGTGTGTTATGAGCGGTCACTTTGATAAAAAGAT
GP108752-1.seq(1>1833)                             →  TAAAACCGTTTTTGCAGGTAGCAGCTGCAATGATATTGTTTGTACCGAACAGTGTGTTATGAGCGGTCACTTTGATAAAAAGAT
G04-F26342-GP108752-1_T7TER.ab1(1>799)             ←  TAAAACCGTTTTTGCAGGTAGCAGCTGCAATGATATTGTTTGTACCGAACAGTGTGTTATGAGCGGTCACTTTGATAAAAAGAT

                                                      1520      1530      1540      1550      1560      1570      1580      1590
                                                      CCGCTTTTGGGATATTCGCAGCGAAAGCATTGTGCGTGAAATGGAACTGCTGGGTAAAATTACAGCACTGGATCTGAATCCGGA
GP108752-1.seq(1>1833)                             →  CCGCTTTTGGGATATTCGCAGCGAAAGCATTGTGCGTGAAATGGAACTGCTGGGTAAAATTACAGCACTGGATCTGAATCCGGA
G04-F26342-GP108752-1_T7TER.ab1(1>799)             ←  CCGCTTTTGGGATATTCGCAGCGAAAGCATTGTGCGTGAAATGGAACTGCTGGGTAAAATTACAGCACTGGATCTGAATCCGGA

                                                      1600      1610      1620      1630      1640      1650      1660      1670      1680
                                                      ACGTACAGAACTGCTGAGCTGTAGCCGTGATGATCTGCTGAAAGTGATTGATTTACGTACCAACGCAATCAAACAGACCTTTAG
GP108752-1.seq(1>1833)                             →  ACGTACAGAACTGCTGAGCTGTAGCCGTGATGATCTGCTGAAAGTGATTGATTTACGTACCAACGCAATCAAACAGACCTTTAG
G04-F26342-GP108752-1_T7TER.ab1(1>799)             ←  ACGTACAGAACTGCTGAGCTGTAGCCGTGATGATCTGCTGAAAGTGATTGATTTACGTACCAACGCAATCAAACAGACCTTTAG

                                                      1690      1700      1710      1720      1730      1740      1750      1760
                                                      CGCACCGGGTTTTAAATGTGGTAGCGATTGGACCCGTGTTGTTTTTAGTCCGGATGGTAGCTATGTTGCAGCAGGTAGCGCAGA
GP108752-1.seq(1>1833)                             →  CGCACCGGGTTTTAAATGTGGTAGCGATTGGACCCGTGTTGTTTTTAGTCCGGATGGTAGCTATGTTGCAGCAGGTAGCGCAGA
G04-F26342-GP108752-1_T7TER.ab1(1>799)             ←  CGCACCGGGTTTTAAATGTGGTAGCGATTGGACCCGTGTTGTTTTTAGTCCGGATGGTAGCTATGTTGCAGCAGGTAGCGCAGA

                                                      1770      1780      1790      1800      1810      1820      1830      1840
                                                      AGGTAGTCTGTATATTTGGAGCGTTCTGACCGGTAAAGTTGAAAAAGTGCTGAGCAAACAGCATAGCAGCAGTATTAATGCAGT
GP108752-1.seq(1>1833)                             →  AGGTAGTCTGTATATTTGGAGCGTTCTGACCGGTAAAGTTGAAAAAGTGCTGAGCAAACAGCATAGCAGCAGTATTAATGCAGT
G04-F26342-GP108752-1_T7TER.ab1(1>799)             ←  AGGTAGTCTGTATATTTGGAGCGTTCTGACCGGTAAAGTTGAAAAAGTGCTGAGCAAACAGCATAGCAGCAGTATTAATGCAGT

                                                      1850      1860      1870      1880      1890      1900      1910      1920      1930
                                                      TGCATGGTCACCGAGCGGTAGTCATGTTGTTAGCGTTGATAAAGGTTGTAAAGCCGTTCTGTGGGCACAGTATTAACTCGAGCA
GP108752-1.seq(1>1833)                             →  TGCATGGTCACCGAGCGGTAGTCATGTTGTTAGCGTTGATAAAGGTTGTAAAGCCGTTCTGTGGGCACAGTATTAACTCGAG
G04-F26342-GP108752-1_T7TER.ab1(1>799)             ←  TGCATGGTCACCGAGCGGTAGTCATGTTGTTAGCGTTGATAAAGGTTGTAAAGCCGTTCTGTGGGCACAGTATTAACTCGAGCA

                                                      1940      1950
                                                      CCACCACCACCACCACTGAGATCCGGC
G04-F26342-GP108752-1_T7TER.ab1(1>799)             ←  CCACCACCACCACCACTGAGATCCGGC
```
